# Supplementary material for: Neural substrates of treatment-resistant schizophrenia and the response to clozapine: A structural MRI study in a clinical setting
Source: PLoS One. 2026 Mar 19;21(3):e0345078. doi: 10.1371/journal.pone.0345078 (PMC13001982; doi:10.1371/journal.pone.0345078)
Supplement: S1 File — (DOCX) [file pone.0345078.s001.docx]

**S1. SUPPLEMENTARY MATERIALS**

**1. Treatment outcomes in the patients treated with CLZ**

**2. Potential effects of MRI scanners within each group**

**3. Comparison of cortical volume between the CLZ responders and non-responders**

1. ***Treatment outcomes in the patients treated with CLZ***

Among the patients treated with CLZ, the psychopathology at the commencement of CLZ treatment was severe, with the mean BPRS total score at 45.00 (± 21.94). At the timepoint of 1 year of CLZ treatment, these patients exhibited significant improvements: the mean GAF score rose from the mean of 24.35 to 45.30 points (p<0.001), and CGI-S score decreased from the mean of 5.80 to 4.60 (p<0.001), and the mean CGI-C score 5.60.

The length of the delay prior to CLZ introduction did not show a significant relationship with the patients' CLZ response measured by ΔGAF (r=0.042, p=0.859), but it showed a significant relationship with the patients' CGI-C scores (r=−0.447, p=0.048). The length of the delay prior to CLZ introduction did not have a significant relationship with any other parameters at baseline (BPRS, GAF, CGI-S) or at 1 year of CLZ treatment (GAF, CGI-S, CLZ dose).

Regarding the response to CLZ, 12 of the 20 patients with TRS were judged as CLZ-responders (ΔGAF ≥20 points; the R-CLZ group) and the other eight patients were classified as non-CLZ-responders (ΔGAF <20 points; the NonR-CLZ group). There were no significant differences in demographic variables between these groups. At baseline, the mean CGI-S score of the R-CLZ patients was significantly higher than that of the NonR-CLZ patients (6.17 vs. 5.25 points respectively, p=0.042). The length of the delay prior to CLZ introduction did not differ significantly between the groups: R-CLZ, 45.91 months vs. NonR-CLZ, 60.23 (p=0.459). At 1 year after the introduction of CLZ treatment, the R-CLZ patients showed significantly higher GAF scores (50.67) compared to the NonR-CLZ patients (37.25, p=0.036). The changes in the efficacy measures were significantly greater in the R-CLZ patients relative to the NonR-CLZ patients: ΔGAF (R-CLZ, 28.17 vs. NonR-CLZ, 10.13 points, p<0.001) and CGI-C (R-CLZ, 5.83 vs. NonR-CLZ, 5.25 points, p=0.028).

1. ***Potential effects of MRI scanners within each group***

Since the present study did not apply harmonization (ComBat) to correct for potential scanner effects, we examined whether difference among MRI scanners could have influenced the findings.

First, in the patient group, whole-brain volume (“BrainSegVol”) appeared unlikely to be influenced by the five MRI scanners (**S1A Fig**). Similarly, in the HC group, there were no clear differences in the brain-volume distributions between the two scanners (**S1B Fig**). However, because the patient and HC groups differed in the MRI scanners used, we further examined whether scanner effects could influence the between-group comparison of brain volume. As an example, we show results for the left rACC **(S2 Fig),** which exhibited the largest difference among the three groups in the volume-ratio analyses (*F*=32.595, p=1.0×10^-10^: **S5 Table**). In the volume-level comparison, HC volume appeared slightly larger than TRS volume, consistent with the statistical significance by three-group ANOVA (*F*=8.580, p<0.001; TRS<HC, p<0.001, Bonferroni corrected *post-hoc* test) (**S2A Fig**). For eTIV, on the other hand, it is likely that the HC had slightly smaller volume than the non-TRS, which was supported by the three-group ANOVA (*F*=10.007, p<0.001; nonTRS>HC, p<0.001, Bonferroni correct *post-hoc* test) (**S2B Fig**). Notably, within the HC group, participants scanned on MRI-7 showed a trend toward smaller volumes. Together, these observations suggest that volume-level comparisons (particularly comparison vs. HCs drawn from an open dataset) could be influenced by scanner differences, because a global trend toward larger volumes in schizophrenia relative to HCs would be unexpected. When we instead analyzed volume ratios, the separation between HC and patient groups became clearer (**S2C Fig**; also see the **Results** section), and the MRI-7-specific trend was no longer apparent. Thus, although scanner-related differences seemed minimal within the patient and HC groups considered separately (**S1 Fig**), scanner effects may still have influenced between-group comparisons. The volume-ratio approach may help attenuate such effects and may better reflect true between-group differences.

1. ***Comparison of cortical volume between the CLZ responders and non-responders***

No significantly different region was observed at the statistical threshold after FDR correction in the multiple comparison. The left rACC, left pars opercularis, right superior frontal, right precentral, and left pericalcarine regions were different at a nominal level of significance, with all of these regions larger in the R-CLZ patients compared to the NonR-CLZ patients (**S6 Table and S3 Fig**). These results were almost the same when the analysis included the antipsychotic dose as a covariate (**S7 Table**).
