# Supplementary figures and images for: Neural substrates of treatment-resistant schizophrenia and the response to clozapine: A structural MRI study in a clinical setting
Source: PLoS One. 2026 Mar 19;21(3):e0345078. doi: 10.1371/journal.pone.0345078 (PMC13001982; doi:10.1371/journal.pone.0345078)

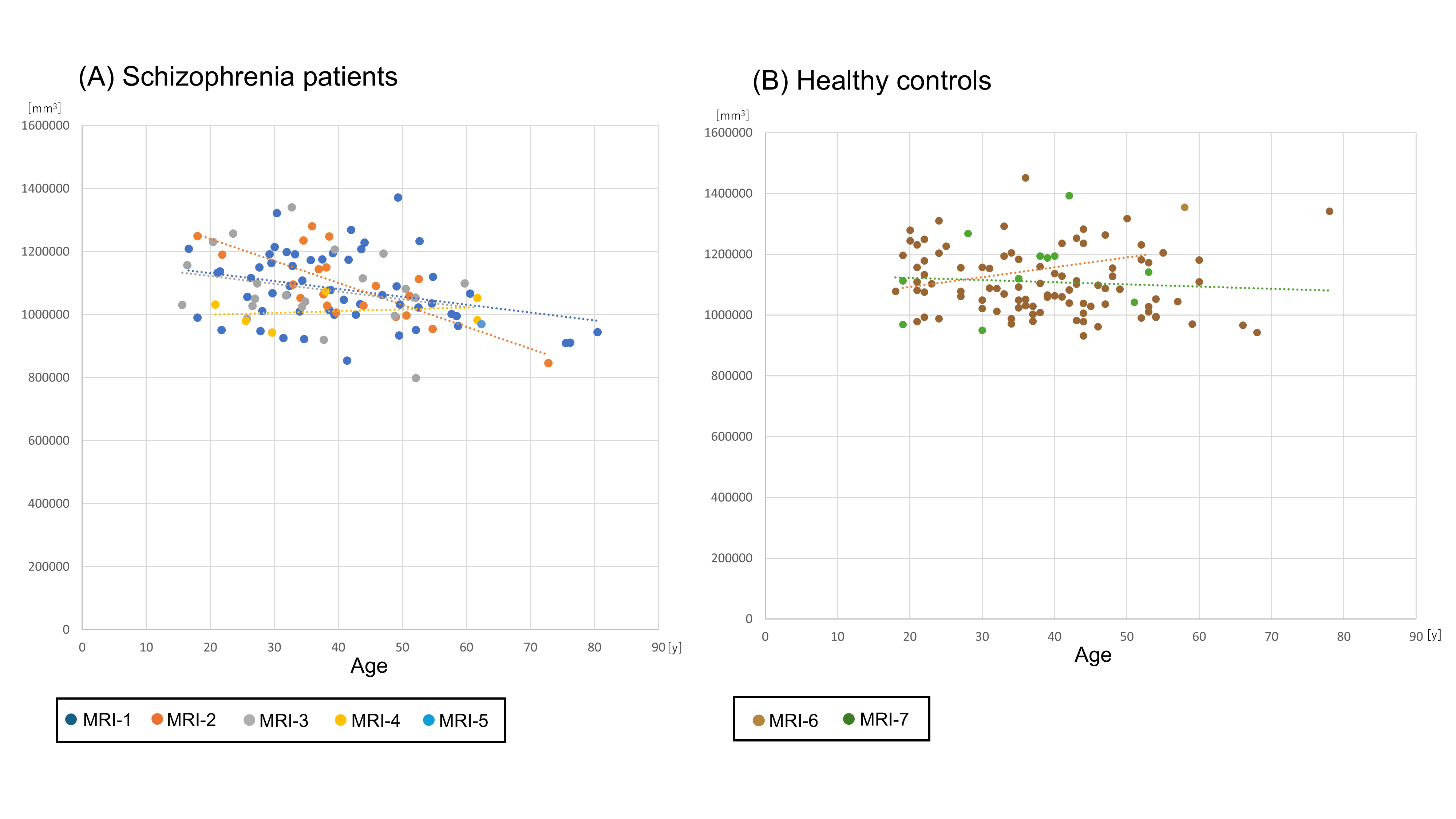

Supplement: S1 Fig — (TIF) [file pone.0345078.s002.TIF]

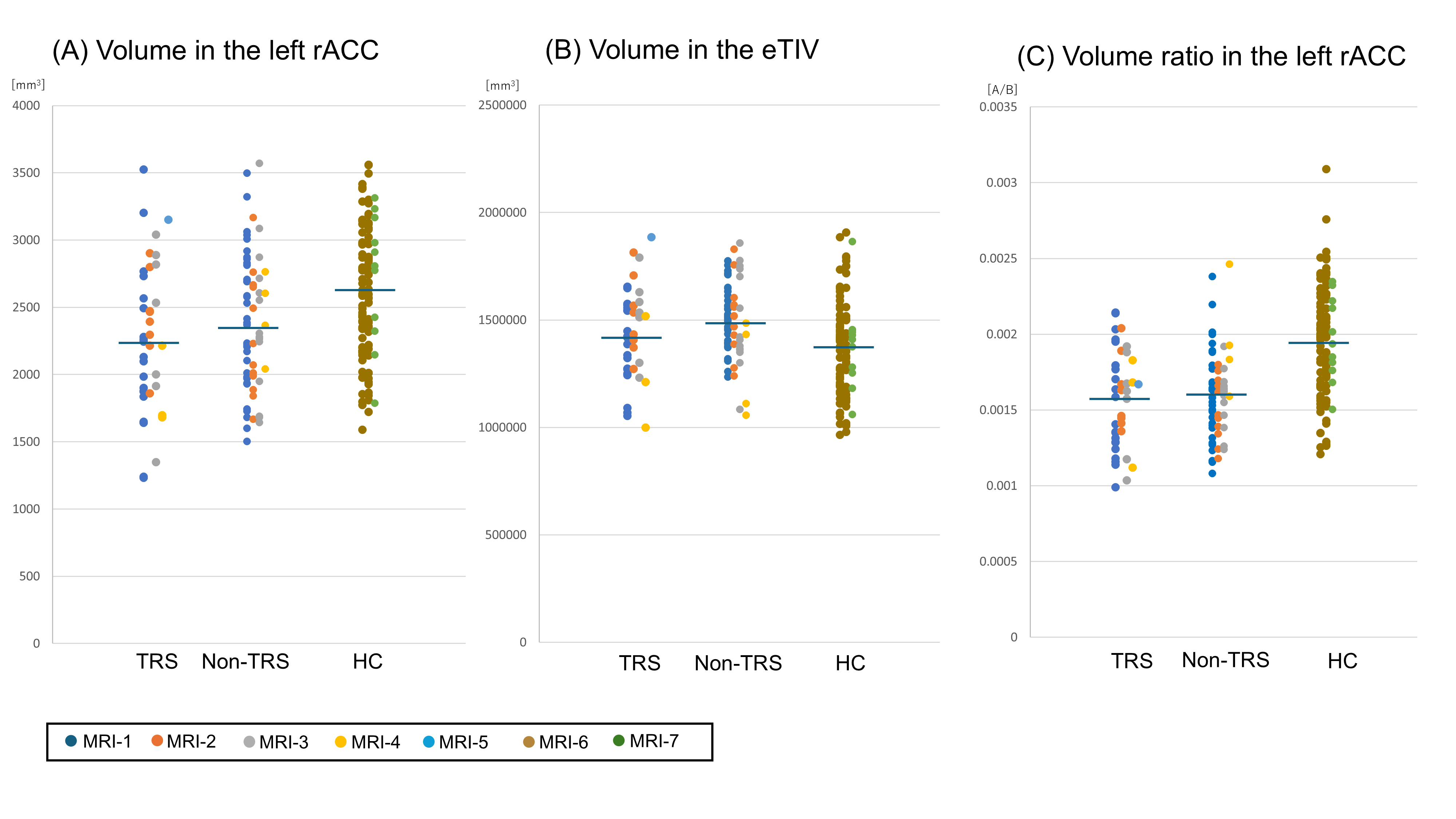

Supplement: S2 Fig — The horizontal line represents the mean of each group. (TIF) [file pone.0345078.s003.Tif]

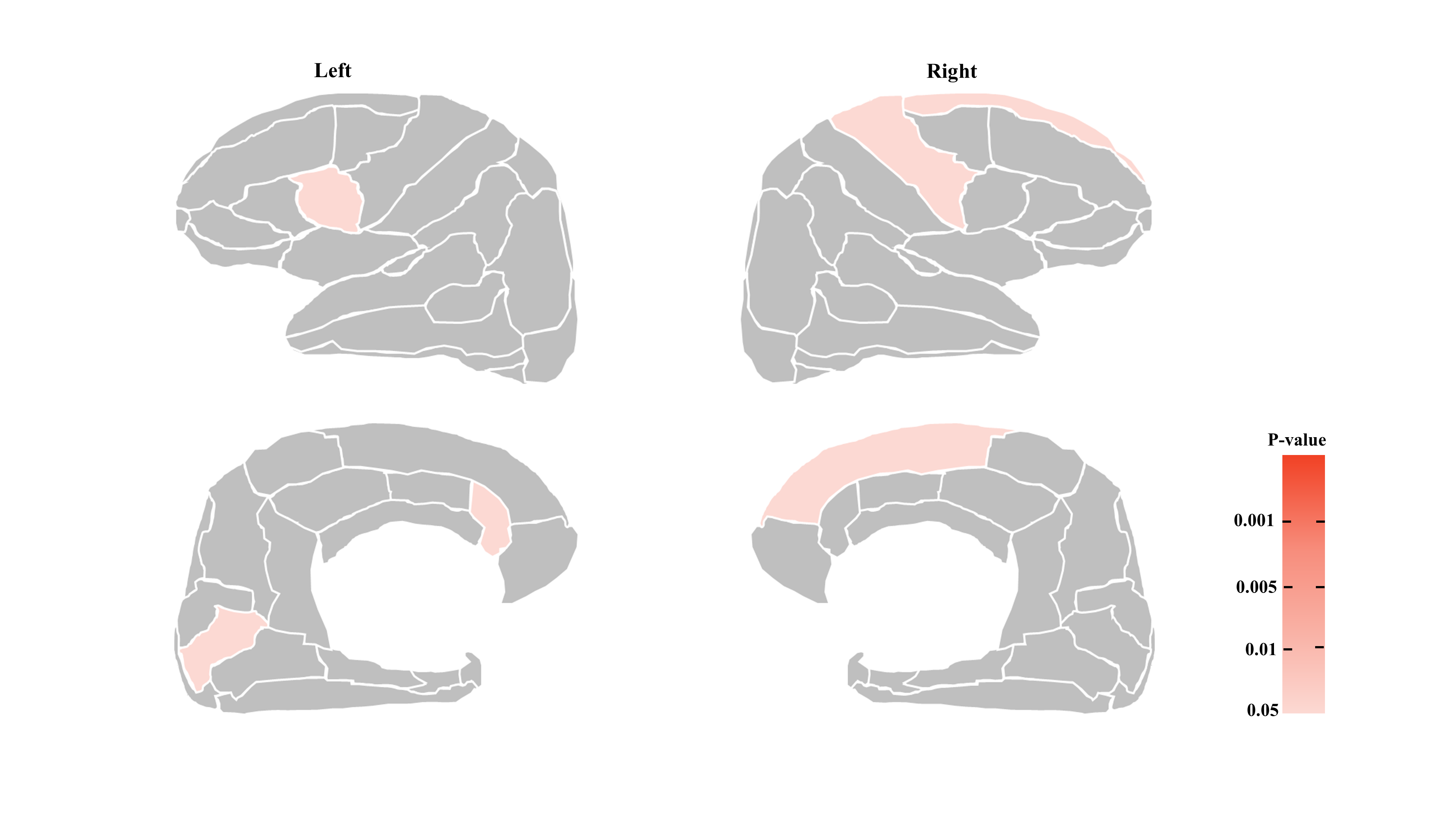

Supplement: S3 Fig — (TIF) [file pone.0345078.s004.TIF]
