# Supplementary material for: Neural substrates of treatment-resistant schizophrenia and the response to clozapine: A structural MRI study in a clinical setting
Source: PLoS One. 2026 Mar 19;21(3):e0345078. doi: 10.1371/journal.pone.0345078 (PMC13001982; doi:10.1371/journal.pone.0345078)
Supplement: S1 Table — (DOCX) [file pone.0345078.s005.docx]

**Suppl. Table S1. The parameters of MRI systems in the present study**

| **Protocol** | **MRI-1** | **MRI-2** | **MRI-3** | **MRI-4** |
| --- | --- | --- | --- | --- |
| **Site** | Chiba Univ. | Chiba Univ. | Chiba Univ. | Chiba Univ. |
| **Scanner** | GE Signa HDxt | GE Discovery 750 | PHILIPS Intera Achieva | PHILIPS Ingenia |
| **Field strength** | 1.5T | 3T | 1.5T | 3T |
| **MR acquisition type** | Sag 3D-IR-T1SPGR | Sag 3D-IR-T1 SPGR | Sg 3D T1FFE | 3D IR-T1 |
| **Voxel size, mm^3^** | 0.9375×0.9375×1.4 | 1×1×1.4 | 0.94×0.94×1.4 | 0.94×1×1.4 |
| **Repetition time, ms** | 7 | 8.2 | 22 | 8 |
| **Echo time, ms** | 3 | 3.3 | 4.6 | 4 |
| **Inversion time, ms** | 420 | 420 | none | 1010.1 |
| **Flip angle, °** | 15 | 15 | 25 | 8 |
| **Field of view, mm^2^** | 240×240 | 256×256 | 240×240 | 240×240 |
| **Image matrix, mm** | 256×256 | 256×256 | 256×256 | 256×240 |
| **Protocol** | **MRI-5** | **MRI-6** | **MRI-7** |  |
| **Site** | Asahi General | Kyoto Univ. | Kyoto Univ. |  |
| **Scanner** | Siemens Avant | Siemens TimTrio | Siemens |  |
| **Field strength** | 1.5T | 3T | 3T |  |
| **MR acquisition type** | T1 | T1 | T1 |  |
| **Voxel size, mm^3^** | 0.8984×0.8984 | 0.9375×0.9375×1.0 | 0.9375×0.9375×1.0 |  |
| **Repetition time, ms** | 1700 | 2000 | 2000 |  |
| **Echo time, ms** | 3.14 | 3.4 | 4.38 |  |
| **Inversion time, ms** | 800 | 990 | 990 |  |
| **Flip angle, °** | 15 | 8 | 8 |  |
| **Field of view, mm^2^** | 230×230 | 225×240 | 225×240 |  |
| **Image matrix, mm** | 256×256 | 240×256 | 240×256 |  |
