# Supplementary material for: Neural substrates of treatment-resistant schizophrenia and the response to clozapine: A structural MRI study in a clinical setting
Source: PLoS One. 2026 Mar 19;21(3):e0345078. doi: 10.1371/journal.pone.0345078 (PMC13001982; doi:10.1371/journal.pone.0345078)
Supplement: S2 Table — (DOCX) [file pone.0345078.s006.docx]

**Suppl. Table S2. The TRS** **patients' demographic and treatment information**

| **Variable** | **CLZ-TRS**  **n=20** | **Non-CLZ-TRS**  **n=20** | **Statistical values** |
| --- | --- | --- | --- |
| **Males/female, n** | 8 / 12 | 8 / 12 | χ^2^=0.00, p=1.00 |
| **Age, yrs** | 40.49 (10.31) | 38.87 (16.05) | *t*=0.380, p=0.706 |
| **Age at illness onset, yrs** | 21.95 (5.81) | 24.53 (11.06) | *t*=－0.925, p=0.363 |
| **Age at TRS establishment, yrs** | 36.25 (10.82) | 41.06 (14.24) | *t*=－0.622, p=0.538 |
| **No. of hospital admissions** | 5.55 (4.55) | 2.55 (4.66) | *t*=2.060, **p=0.046** |
| **Antipsychotic dose at MRI examination (CP-eq.), mg/day** | 1191.04 (626.06) | 497.77 (366.07) | *t*=4.275, **p<0.001** |
| **Delay prior to CLZ introduction, months** | 51.64 (40.94) | – | – |
| ***Measurements at CLZ introduction*** | | | |
| **BPRS** | 45.00 (21.94) | – | – |
| **GAF** | 24.35 (10.07) | – | – |
| **CGI-S** | 5.80 (1.01) | – | – |
| ***Measurements at 1 y of CLZ treatment*** | | | |
| **CLZ dose, mg/day** | 387.50 (153.58) | – | – |
| **GAF** | 45.30 (14.29) | – | *t*=–7.649, **p<0.001*** |
| **CGI-S** | 4.60 (1.05) | – | *t*=4.485, **p<0.001*** |
| **ΔGAF** | 20.95 (12.25) | – | – |
| **CGI-C** | 5.60 (0.60) | – | – |

*: compared to the score at the CLZ introduction

BPRS: Brief Psychiatric Rating Scale, CGI-C: Clinical Global Impressions-Change scale, CGI-S: Clinical Global Impressions-Severity of illness scale, CLZ: clozapine, GAF: Global Assessment of Functioning scale
