# Supplementary material for: Neural substrates of treatment-resistant schizophrenia and the response to clozapine: A structural MRI study in a clinical setting
Source: PLoS One. 2026 Mar 19;21(3):e0345078. doi: 10.1371/journal.pone.0345078 (PMC13001982; doi:10.1371/journal.pone.0345078)
Supplement: S3 Table — (DOCX) [file pone.0345078.s007.docx]

**Suppl. Table S3. The comparison of cortical volume and volume ratio between the TRS and nonTRS groups**

| **Lobe** | **Region** | **Volume** [mm^3^] | | **Volume ratio** | | **Volume** | | **Post-hoc** | **Cohen's d** |
| --- | --- | --- | --- | --- | --- | --- | --- | --- | --- |
|  |  | **TRS** | **Non-TRS** | ***F*-value** | **p-value** | ***F*-value** | **p-value** |  |  |
| Left eTIV |  | 1406252.540 | 1493326.941 |  |  |  |  |  |  |
| Left frontal | Caudal anterior cingulate | 1390.095 | 1630.609 | 3.409 | 0.068 | 4.960 | **0.028** | TRS < nonTRS | 0.446 |
|  | Rostral anterior cingulate | 2249.048 | 2390.578 | 0.547 | 0.461 | 0.969 | 0.327 |  | 0.272 |
|  | Medial orbitofrontal | 5089.548 | 5310.578 | 0.055 | 0.816 | 2.963 | 0.088 |  | 0.314 |
|  | Frontal pole | 1013.357 | 974.188 | 4.610 | **0.034** | 0.474 | 0.493 | TRS > nonTRS | (0.190) |
|  | Lateral orbitofrontal | 7030.690 | 7375.172 | 0.277 | 0.600 | 3.866 | 0.052 |  | 0.349 |
|  | Pars orbitalis | 2173.095 | 2244.328 | 0.000 | 1.000 | 1.193 | 0.277 |  | 0.195 |
|  | Pars triangularis | 3520.214 | 3552.359 | 1.206 | 0.275 | 0.056 | 0.813 |  | 0.048 |
|  | Pars opercularis | 4256.262 | 4360.156 | 0.066 | 0.798 | 0.412 | 0.522 |  | 0.113 |
|  | Rostral middle frontal | 13973.357 | 15099.750 | 3.082 | 0.082 | 7.605 | **0.007** | TRS < nonTRS | 0.452 |
|  | Caudal middle frontal | 5555.476 | 6003.375 | 1.186 | 0.279 | 4.674 | **0.033** | TRS < nonTRS | 0.405 |
|  | Superior frontal | 20815.833 | 21696.656 | 0.062 | 0.804 | 3.399 | 0.068 |  | 0.293 |
|  | Precentral | 12890.333 | 13236.609 | 0.208 | 0.649 | 1.053 | 0.307 |  | 0.200 |
|  | Paracentral | 3371.119 | 3415.219 | 0.459 | 0.500 | 0.319 | 0.573 |  | 0.097 |
|  | Insula | 6526.881 | 6842.781 | 0.151 | 0.699 | 3.273 | 0.073 |  | 0.367 |
| Left temporal | Bank of STS | 2085.976 | 2269.125 | 2.301 | 0.133 | 4.991 | **0.028** | TRS < nonTRS | 0.419 |
|  | Transverse temporal | 1112.167 | 1183.266 | 0.386 | 0.536 | 2.802 | 0.097 |  | 0.355 |
|  | Superior temporal | 11452.976 | 12024.422 | 0.230 | 0.632 | 4.626 | **0.034** | TRS < nonTRS | 0.362 |
|  | Middle temporal | 9944.143 | 10729.375 | 3.054 | 0.084 | 5.832 | **0.018** | TRS < nonTRS | 0.465 |
|  | Inferior temporal | 10403.905 | 10967.047 | 0.512 | 0.476 | 3.322 | 0.071 |  | 0.363 |
|  | Temporal pole | 2600.429 | 2721.125 | 0.001 | 0.971 | 1.331 | 0.251 |  | 0.256 |
|  | Entorhinal | 1756.429 | 1807.984 | 0.183 | 0.670 | 0.191 | 0.663 |  | 0.118 |
|  | Parahippocampal | 1830.524 | 1905.406 | 0.015 | 0.904 | 2.260 | 0.136 |  | 0.283 |
|  | Fusiform | 8718.690 | 9196.094 | 0.426 | 0.516 | 3.720 | 0.057 |  | 0.384 |
| Left parietal | Postcentral | 9205.357 | 9525.141 | 0.028 | 0.868 | 2.116 | 0.149 |  | 0.267 |
|  | Superior parietal | 12752.143 | 12943.281 | 0.546 | 0.462 | 0.383 | 0.537 |  | 0.109 |
|  | Inferior parietal | 11555.024 | 12073.516 | 0.049 | 0.826 | 1.462 | 0.229 |  | 0.285 |
|  | Supramarginal | 10900.500 | 11037.828 | 0.697 | 0.406 | 0.106 | 0.745 |  | 0.077 |
|  | Isthmus | 2519.000 | 2541.891 | 1.420 | 0.236 | 0.001 | 0.978 |  | 0.056 |
|  | Post cingulate | 2808.738 | 2985.156 | 0.444 | 0.507 | 2.522 | 0.115 |  | 0.370 |
|  | Precuneus | 9187.833 | 9402.844 | 0.155 | 0.694 | 0.618 | 0.434 |  | 0.163 |
| Left occipital | Lateral occipital | 11210.429 | 11656.313 | 0.017 | 0.898 | 1.472 | 0.228 |  | 0.252 |
|  | Lingual | 6006.143 | 6378.469 | 0.221 | 0.640 | 3.283 | 0.073 |  | 0.356 |
|  | Cuneus | 2869.738 | 2964.594 | 0.060 | 0.807 | 1.089 | 0.299 |  | 0.203 |
|  | Pericalcarine | 1888.690 | 1988.297 | 0.139 | 0.710 | 1.580 | 0.212 |  | 0.258 |
| Right eTIV |  | 1406252.540 | 1493326.941 |  |  |  |  |  |  |
| Right frontal | Caudal anterior cingulate | 1852.381 | 1914.203 | 0.015 | 0.903 | 0.202 | 0.654 |  | 0.123 |
|  | Rostral anterior cingulate | 1695.452 | 1853.938 | 0.809 | 0.371 | 2.498 | 0.117 |  | 0.351 |
|  | Medial orbitofrontal | 5370.643 | 5631.656 | 0.526 | 0.470 | 3.731 | 0.056 |  | 0.366 |
|  | Frontal pole | 1211.548 | 1249.313 | 0.058 | 0.810 | 1.059 | 0.306 |  | 0.185 |
|  | Lateral orbitofrontal | 7084.405 | 7379.172 | 0.045 | 0.833 | 3.028 | 0.085 |  | 0.291 |
|  | Pars orbitalis | 2616.810 | 2723.203 | 0.079 | 0.779 | 1.722 | 0.192 |  | 0.223 |
|  | Pars triangularis | 4229.595 | 4181.703 | 3.028 | 0.085 | 0.145 | 0.704 |  | (0.058) |
|  | Pars opercularis | 3629.833 | 3602.359 | 2.933 | 0.090 | 0.278 | 0.599 |  | (0.041) |
|  | Rostral middle frontal | 14470.714 | 15449.234 | 0.716 | 0.400 | 6.56 | **0.012** | TRS < nonTRS | 0.391 |
|  | Caudal middle frontal | 5521.262 | 5751.109 | 0.009 | 0.923 | 1.468 | 0.229 |  | 0.233 |
|  | Superior frontal | 19289.048 | 20258.406 | 0.279 | 0.599 | 5.589 | **0.02** | TRS < nonTRS | 0.365 |
|  | Precentral | 12605.214 | 12916.609 | 0.139 | 0.710 | 0.844 | 0.36 |  | 0.182 |
|  | Paracentral | 3637.524 | 3572.313 | 3.627 | 0.060 | 0.69 | 0.408 |  | (0.121) |
|  | Insula | 6360.762 | 6617.813 | 0.003 | 0.958 | 1.667 | 0.200 |  | 0.280 |
| Right temporal | Bank of STS | 1838.595 | 1996.0938 | 2.114 | 0.149 | 4.906 | **0.029** | TRS < nonTRS | 0.456 |
|  | Transverse temporal | 843.262 | 919.078 | 1.715 | 0.193 | 6.445 | **0.013** | TRS < nonTRS | 0.469 |
|  | Superior temporal | 11118.786 | 11744.406 | 0.330 | 0.567 | 4.73 | **0.032** | TRS < nonTRS | 0.389 |
|  | Middle temporal | 10869.738 | 11680.922 | 2.399 | 0.125 | 8.057 | **0.005** | TRS < nonTRS | 0.501 |
|  | Inferior temporal | 9954.357 | 10557.063 | 0.692 | 0.407 | 3.747 | 0.056 |  | 0.354 |
|  | Temporal pole | 2670.357 | 2670.109 | 1.025 | 0.314 | 0.069 | 0.793 |  | (0.001) |
|  | Entorhinal | 1704.548 | 1674.344 | 0.067 | 0.796 | 0.367 | 0.546 |  | (0.076) |
|  | Parahippocampal | 1714.024 | 1767.828 | 0.058 | 0.810 | 0.617 | 0.434 |  | 0.210 |
|  | Fusiform | 8417.262 | 8867.375 | 0.119 | 0.731 | 3.884 | 0.052 |  | 0.348 |
| Right parietal | Postcentral | 8803.548 | 9235.875 | 0.176 | 0.675 | 3.277 | 0.073 |  | 0.329 |
|  | Superior parietal | 12436.286 | 12794.016 | 0.033 | 0.856 | 1.116 | 0.293 |  | 0.210 |
|  | Inferior parietal | 13640.500 | 14331.250 | 0.385 | 0.536 | 3.51 | 0.064 |  | 0.334 |
|  | Supramarginal | 9360.810 | 9679.281 | 0.007 | 0.934 | 0.794 | 0.375 |  | 0.208 |
|  | Isthmus | 2389.333 | 2438.766 | 0.427 | 0.515 | 0.264 | 0.608 |  | 0.128 |
|  | Post cingulate | 2903.738 | 3067.594 | 0.220 | 0.640 | 1.642 | 0.203 |  | 0.298 |
|  | Precuneus | 9581.643 | 9713.969 | 0.795 | 0.375 | 0.235 | 0.629 |  | 0.099 |
| Right occipital | Lateral occipital | 11239.595 | 11783.078 | 0.009 | 0.926 | 4.238 | **0.042** | TRS < nonTRS | 0.348 |
|  | Lingual | 6485.238 | 6773.031 | 0.000 | 0.994 | 1.318 | 0.254 |  | 0.246 |
|  | Cuneus | 3102.095 | 3378.484 | 2.840 | 0.095 | 7.643 | **0.007** | TRS < nonTRS | 0.508 |
|  | Pericalcarine | 2169.286 | 2288.484 | 0.253 | 0.616 | 1.341 | 0.25 |  | 0.248 |

eTIV: estimated intracranial volume, STS: superior temporal sulcus.
