# Supplementary material for: Neural substrates of treatment-resistant schizophrenia and the response to clozapine: A structural MRI study in a clinical setting
Source: PLoS One. 2026 Mar 19;21(3):e0345078. doi: 10.1371/journal.pone.0345078 (PMC13001982; doi:10.1371/journal.pone.0345078)
Supplement: S4 Table — (DOCX) [file pone.0345078.s008.docx]

**Suppl. Table S4. The comparison of cortical volume between the TRS and nonTRS groups in the analysis including age at illness onset and**

**antipsychotic dose as additional covariates**

| **Lobe** | **Region** | **Left** | | | **Right** | | |
| --- | --- | --- | --- | --- | --- | --- | --- |
|  |  | ***F*-value** | **p-value** | **Post-hoc** | ***F*-value** | **p-value** | **Post-hoc** |
|  | eTIV | 0.826 | 0.366 |  |  |  |  |
| Frontal | Caudal anterior cingulate | 4.778 | **0.031** | TRS < nonTRS | 0.234 | 0.629 |  |
|  | Rostral anterior cingulate | 1.345 | 0.249 |  | 1.6 | 0.209 |  |
|  | Medial orbitofrontal | 1.624 | 0.206 |  | 1.84 | 0.178 |  |
|  | Frontal pole | 3.998 | **0.048** | TRS > nonTRS | 0.077 | 0.781 |  |
|  | Lateral orbitofrontal | 2.234 | 0.138 |  | 1.108 | 0.295 |  |
|  | Pars orbitalis | 0.179 | 0.673 |  | 1.367 | 0.245 |  |
|  | Pars triangularis | 0.738 | 0.392 |  | 1.231 | 0.27 |  |
|  | Pars opercularis | 0.629 | 0.430 |  | 0.43 | 0.514 |  |
|  | Rostral middle frontal | 4.459 | **0.037** | TRS < nonTRS | 4.553 | **0.035** | TRS < nonTRS |
|  | Caudal middle frontal | 3.998 | **0.049** | TRS < nonTRS | 1.706 | 0.195 |  |
|  | Superior frontal | 1.492 | 0.225 |  | 3.058 | 0.064 |  |
|  | Precentral | 1.185 | 0.279 |  | 1.63 | 0.205 |  |
|  | Paracentral | 0.338 | 0.562 |  | 1.291 | 0.259 |  |
|  | Insula | 1.509 | 0.222 |  | 0.756 | 0.387 |  |
| Temporal | Bank of STS | 3.455 | 0.066 |  | 2.999 | 0.086 |  |
|  | Transverse temporal | 2.346 | 0.129 |  | 5.603 | **0.020** | TRS < nonTRS |
|  | Superior temporal | 3.222 | 0.076 |  | 2.359 | 0.128 |  |
|  | Middle temporal | 5.240 | **0.024** | TRS < nonTRS | 6.743 | **0.011** | TRS < nonTRS |
|  | Inferior temporal | 0.477 | 0.491 |  | 0.8 | 0.373 |  |
|  | Temporal pole | 0.172 | 0.679 |  | 0.295 | 0.588 |  |
|  | Entorhinal | 0.414 | 0.521 |  | 2.277 | 0.135 |  |
|  | Parahippocampal | 0.311 | 0.578 |  | 0.292 | 0.59 |  |
|  | Fusiform | 1.078 | 0.302 |  | 1.833 | 0.179 |  |
| Parietal | Postcentral | 2.269 | 0.135 |  | 1.987 | 0.162 |  |
|  | Superior parietal | 0.100 | 0.752 |  | 0.642 | 0.425 |  |
|  | Inferior parietal | 0.520 | 0.472 |  | 3.365 | 0.070 |  |
|  | Supramarginal | 0.456 | 0.501 |  | 0.311 | 0.578 |  |
|  | Isthmus | 0.006 | 0.941 |  | 0.045 | 0.833 |  |
|  | Post cingulate | 1.549 | 0.216 |  | 0.081 | 0.777 |  |
|  | Precuneus | 0.320 | 0.573 |  | 0.573 | 0.451 |  |
| Occipital | Lateral occipital | 0.325 | 0.570 |  | 2.358 | 0.128 |  |
|  | Lingual | 2.383 | 0.126 |  | 0.664 | 0.417 |  |
|  | Cuneus | 1.306 | 0.256 |  | 6.101 | **0.015** | TRS < nonTRS |
|  | Pericalcarine | 1.643 | 0.203 |  | 1.3 | 0.257 |  |

eTIV: estimated intracranial volume, STS: superior temporal sulcus.

The analysis dealt age, sex, MRI scanner, age at illness onset and antipsychotic dose with covariates.
