# Supplementary material for: Neural substrates of treatment-resistant schizophrenia and the response to clozapine: A structural MRI study in a clinical setting
Source: PLoS One. 2026 Mar 19;21(3):e0345078. doi: 10.1371/journal.pone.0345078 (PMC13001982; doi:10.1371/journal.pone.0345078)
Supplement: S5 Table — (DOCX) [file pone.0345078.s009.docx]

**Suppl. Table S5. Comparison of cortical volume ratio among the TRS, non-TRS, and HC groups**

| **Lobe** | **Region** | ***F*** | **P*_uncor_*** | **P*_cor_*** | **TRS vs. HC** | | **NonTRS vs. HC** | | **TRS vs. NonTRS** | |
| --- | --- | --- | --- | --- | --- | --- | --- | --- | --- | --- |
|  |  |  |  |  | **p-value** | **Cohen's d** | **p-value** | **Cohen's d** | **p-value** | **Cohen's d** |
| Left frontal | Caudal anterior cingulate | 11.378 | **2.06e-5** | **5.19e-5** | **0.000** | 0.820 | **0.012** | 0.430 | 0.215 | 0.366 |
|  | Rostral anterior cingulate | 32.595 | **<1e-10** | **<1e-10** | **0.000** | 0.988 | **0.000** | 0.946 | 1.000 | 0.103 |
|  | Medial orbitofrontal | 13.398 | **3.35e-6** | **1.04e-5** | **0.000** | 0.629 | **0.000** | 0.664 | 1.000 | (0.012) |
|  | Frontal pole | 13.482 | **3.12e-6** | **1.01e-5** | 0.130 | 0.379 | **0.000** | 0.787 | 0.082 | (0.421) |
|  | Lateral orbitofrontal | 13.098 | **4.38e-6** | **1.29e-5** | **0.000** | 0.616 | **0.000** | 0.656 | 1.000 | 0.029 |
|  | Pars orbitalis | 8.308 | **3.37e-4** | **6.00e-4** | **0.010** | 0.486 | **0.001** | 0.571 | 1.000 | (0.046) |
|  | Pars triangularis | 7.646 | **6.24e-4** | **9.65e-4** | 0.131 | 0.361 | **0.001** | 0.598 | 0.754 | (0.239) |
|  | Pars opercularis | 10.030 | **6.92e-5** | **1.35e-4** | **0.006** | 0.538 | **0.000** | 0.616 | 1.000 | (0.086) |
|  | Rostral middle frontal | 14.819 | **9.62e-7** | **3.63e-6** | **0.000** | 0.762 | **0.000** | 0.586 | 0.477 | 0.251 |
|  | Caudal middle frontal | 5.472 | **4.83e-3** | **6.08e-3** | **0.010** | 0.514 | 0.065 | 0.388 | 1.000 | 0.158 |
|  | Superior frontal | 11.656 | **1.59e-5** | **4.15e-5** | **0.001** | 0.569 | **0.000** | 0.638 | 1.000 | (0.015) |
|  | Precentral | 10.857 | **3.27e-5** | **7.66e-5** | **0.009** | 0.510 | **0.000** | 0.679 | 1.000 | (0.153) |
|  | Paracentral | 16.366 | **2.50e-7** | **1.30e-6** | **0.001** | 0.609 | **0.000** | 0.794 | 1.000 | (0.188) |
|  | Insula | 27.886 | **<1e-10** | **3.13e-10** | **0.000** | 0.851 | **0.000** | 0.894 | 1.000 | 0.022 |
| Left temporal | Bank of STS | 17.134 | **5.06e-5** | **1.04e-4** | **0.000** | 0.809 | **0.000** | 0.674 | 0.727 | 0.232 |
|  | Transverse temporal | 10.542 | **4.35e-5** | **9.53e-5** | **0.001** | 0.603 | **0.001** | 0.578 | 1.000 | 0.091 |
|  | Superior temporal | 15.577 | **4.94e-7** | **2.40e-6** | **0.000** | 0.680 | **0.000** | 0.673 | 1.000 | 0.053 |
|  | Middle temporal | 23.451 | **7.00e-10** | **6.42e-9** | **0.000** | 0.926 | **0.000** | 0.701 | 0.458 | 0.267 |
|  | Inferior temporal | 10.503 | **4.50e-5** | **9.57e-5** | **0.001** | 0.616 | **0.001** | 0.559 | 1.000 | 0.103 |
|  | Temporal pole | 0.215 | 0.807 | 0.831 | – | (0.082) | – | (0.069) | – | (0.013) |
|  | Entorhinal | 3.430 | **0.0342** | **0.0423** | 0.090 | 0.213 | 0.121 | 0.331 | 1.000 | (0.078) |
|  | Parahippocampal | 8.123 | **4.00e-4** | **6.48e-4** | **0.008** | 0.501 | **0.002** | 0.547 | 1.000 | (0.003) |
|  | Fusiform | 10.184 | **6.02e-5** | **1.20e-4** | **0.001** | 0.595 | **0.001** | 0.563 | 1.000 | 0.100 |
| Left parietal | Postcentral | 7.714 | **5.86e-4** | **9.26e-4** | **0.020** | 0.446 | **0.001** | 0.564 | 1.000 | (0.083) |
|  | Superior parietal | 2.444 | 0.089 | 0.103 | – | 0.210 | – | 0.353 | – | (0.164) |
|  | Inferior parietal | 13.706 | **2.55e-6** | **8.68e-6** | **0.000** | 0.635 | **0.000** | 0.646 | 1.000 | 0.006 |
|  | Supramarginal | 6.356 | **2.09e-3** | **2.73e-3** | 0.134 | 0.341 | **0.002** | 0.564 | 1.000 | (0.197) |
|  | Isthmus | 7.640 | **6.27e-4** | **9.48e-4** | 0.156 | 0.351 | **0.001** | 0.603 | 0.659 | (0.269) |
|  | Post cingulate | 15.473 | **5.44e-7** | **2.31e-6** | **0.000** | 0.734 | **0.000** | 0.697 | 1.000 | 0.093 |
|  | Precuneus | 8.372 | **3.18e-4** | **5.40e-4** | **0.027** | 0.443 | **0.001** | 0.586 | 1.000 | (0.157) |
| Left occipital | Lateral occipital | 2.026 | 0.134 | 0.147 | – | 0.281 | – | 0.333 | – | (0.054) |
|  | Lingual | 2.166 | 0.117 | 0.131 | – | 0.333 | – | 0.255 | – | 0.097 |
|  | Cuneus | 3.325 | **0.038** | **0.046** | 0.267 | 0.350 | 0.055 | 0.439 | 1.000 | (0.078) |
|  | Pericalcarine | 0.040 | 0.960 | 0.960 | – | 0.071 | – | 0.032 | – | 0.049 |
| Right frontal | Caudal anterior cingulate | 12.699 | **6.26e-6** | **1.77e-5** | **0.001** | 0.651 | **0.000** | 0.673 | 1.000 | (0.049) |
|  | Rostral anterior cingulate | 18.711 | **3.35e-8** | **2.53e-7** | **2.34e-6** | 0.823 | **7.69e-6** | 0.709 | 1.000 | 0.174 |
|  | Medial orbitofrontal | 27.112 | **<1e-10** | **4.62e-10** | **1.16e-4** | 0.840 | **3.38e-8** | 0.848 | 1.000 | 0.050 |
|  | Frontal pole | 2.321 | 0.101 | 0.114 | – | 0.264 | – | 0.354 | – | (0.093) |
|  | Lateral orbitofrontal | 12.426 | **7.96e-6** | **2.17e-5** | **7.81e-4** | 0.585 | **6.76e-5** | 0.660 | 1.000 | (0.032) |
|  | Pars orbitalis | 6.725 | **1.48e-3** | **2.01e-3** | **0.015** | 0.479 | **7.00e-3** | 0.492 | 1.000 | (0.001) |
|  | Pars triangularis | 15.464 | **5.46e-7** | **2.18e-6** | **0.0114** | 0.475 | **5.01e-7** | 0.796 | 0.341 | (0.343) |
|  | Pars opercularis | 17.551 | **9.05e-8** | **5.60e-7** | **2.97e-3** | 0.555 | **1.18e-7** | 0.807 | 0.450 | (0.347) |
|  | Rostral middle frontal | 16.559 | **2.11e-7** | **1.20e-6** | **1.2e-5** | 0.732 | **2.10e-5** | 0.715 | 1.000 | 0.116 |
|  | Caudal middle frontal | 6.086 | **2.70e-3** | **3.46e-3** | **0.0325** | 0.440 | **7.55e-3** | 0.499 | 1.000 | (0.035) |
|  | Superior frontal | 18.359 | **4.51e-8** | **3.07e-7** | **1.34e-5** | 0.728 | **2.13e-6** | 0.757 | 1.000 | 0.038 |
|  | Precentral | 6.596 | **1.67e-3** | **2.22e-3** | 0.063 | 0.391 | **2.54e-3** | 0.538 | 1.000 | (0.142) |
|  | Paracentral | 19.422 | **1.84e-8** | **1.56e-7** | **4.48e-3** | 0.538 | **1.64e-8** | 0.888 | 0.197 | (0.422) |
|  | Insula | 27.030 | **<1e-10** | **4.11e-10** | **4.15e-7** | 0.837 | **2.79e-9** | 0.902 | 1.000 | (0.036) |
| Right temporal | Bank of STS | 30.774 | **<1e-10** | **<1e-10** | **0.000** | 0.997 | **0.000** | 0.842 | 0.676 | 0.245 |
|  | Transverse temporal | 7.514 | **7.06e-4** | **1.04e-3** | **1.55e-3** | 0.570 | **0.0258** | 0.442 | 0.768 | 0.216 |
|  | Superior temporal | 13.838 | **2.27e-6** | **8.12e-6** | **8.42e-5** | 0.670 | **9.18e-5** | 0.649 | 1.000 | 0.094 |
|  | Middle temporal | 29.723 | **<1e-10** | **<1e-10** | **9.27e-10** | 0.980 | **5.13e-8** | 0.807 | 0.461 | 0.261 |
|  | Inferior temporal | 8.793 | **2.16e-4** | **3.86e-4** | **1.07e-3** | 0.621 | **5.73e-3** | 0.539 | 1.000 | 0.094 |
|  | Temporal pole | 0.987 | 0.374 | 0.398 | – | 0.01 | – | 0.281 | – | (0.260) |
|  | Entorhinal | 8.491 | **2.86e-4** | **4.98e-4** | **0.0166** | 0.170 | **6.92e-4** | 0.569 | 1.000 | (0.291) |
|  | Parahippocampal | 6.868 | **1.29e-3** | **1.83e-3** | **0.0308** | 0.421 | **2.99e-3** | 0.507 | 1.000 | (0.073) |
|  | Fusiform | 15.534 | **5.13e-7** | **2.33e-6** | **0.000** | 0.710 | **0.000** | 0.728 | 1.000 | 0.055 |
| Right parietal | Postcentral | 11.180 | **2.44e-5** | **5.92e-5** | **8.44e-4** | 0.599 | **2.89e-4** | 0.606 | 1.000 | 0.0368 |
|  | Supeior parietal | 0.358 | 0.699 | 0.731 | – | 0.092 | – | 0.155 | – | (0.069) |
|  | Inferior parietal | 8.838 | **2.07e-4** | **3.81e-4** | **2.21e-3** | 0.570 | **2.36e-3** | 0.535 | 1.000 | 0.076 |
|  | Supramarginal | 6.725 | **1.48e-3** | **2.05e-3** | **0.0220** | 0.454 | **4.62e-3** | 0.493 | 1.000 | (0.035) |
|  | Isthmus | 7.274 | **8.83e-4** | **1.28e-3** | 0.0571 | 0.402 | **1.23e-3** | 0.576 | 1.000 | (0.160) |
|  | Post cingulate | 10.771 | **3.53e-5** | **8.00e-5** | **9.31e-4** | 0.637 | **4.43e-4** | 0.609 | 1.000 | 0.045 |
|  | Precuneus | 9.214 | **1.46e-4** | **2.76e-4** | **0.0346** | 0.427 | **1.87e-4** | 0.625 | 1.000 | (0.227) |
| Right occipital | Lateral occipital | 1.538 | 0.217 | 0.235 | – | 0.273 | – | 0.288 | – | 0.015 |
|  | Lingual | 2.673 | 0.071 | 0.084 | – | 0.320 | – | 0.327 | – | (0.013) |
|  | Cuneus | 3.126 | **0.046** | 0.055 | **0.0399** | 0.403 | 1 | 0.166 | 0.359 | 0.297 |
|  | Pericalcarine | 0.080 | 0.923 | 0.936 | – | 0.046 | – | (0.013) | – | 0.068 |

Cohen's d with parentheses indicates negative value. STS: superior temporal sulcus.
