# Supplementary material for: Neural substrates of treatment-resistant schizophrenia and the response to clozapine: A structural MRI study in a clinical setting
Source: PLoS One. 2026 Mar 19;21(3):e0345078. doi: 10.1371/journal.pone.0345078 (PMC13001982; doi:10.1371/journal.pone.0345078)
Supplement: S6 Table — (DOCX) [file pone.0345078.s010.docx]

**Suppl. Table 6. Comparison of cortical volume ratios between the CLZ responders and CLZ non-responders**

| **Region** | **CLZ responders**  **(n=12)** | **CLZ non-responders (n=8)** | **Statistic values^#1^** | **Statistic values^#2^** |
| --- | --- | --- | --- | --- |
| Lt rostral anterior cingulate | 0.001685 (0.000289) | 0.001512 (0.000299) | *F*=7.586, **p=0.015** | *F*=6.879, **p=0.020** |
| Lt pars opercularis | 0.003096 (0.000535) | 0.002667 (0.000299) | *F*=6.584, **p=0.022** | *F*=6.068, **p=0.027** |
| Rt superior frontal | 0.01461 (0.00216) | 0.01454 (0.00159) | *F*=4.961, **p=0.042** | *F*=4.456, p=0.053 |
| Rt precentral | 0.009127 (0.001629) | 0.008761 (0.000580) | *F*=5.155, **p=0.038** | *F*=5.750, **p=0.031** |
| Lt pericalcarine | 0.001400 (0.000343) | 0.001270 (0.000209) | *F*=6.485, **p=0.022** | *F*=6,282, **p=0.025** |

Only the regions with significant differences are shown.

#1. Controlling for age, sex and MRI scanner

#2. Controlling for age, sex, MRI scanner and antipsychotic dose
