# Supplementary material for: Neural substrates of treatment-resistant schizophrenia and the response to clozapine: A structural MRI study in a clinical setting
Source: PLoS One. 2026 Mar 19;21(3):e0345078. doi: 10.1371/journal.pone.0345078 (PMC13001982; doi:10.1371/journal.pone.0345078)
Supplement: S7 Table — (DOCX) [file pone.0345078.s011.docx]

**Suppl. Table S7. Cortical areas potentially relevant to the CLZ response in the analysis including　antipsychotic dose as additional covariate**

| **Region** | **Clinical parameters** | **Statistic values** |
| --- | --- | --- |
| Lt caudal anterior cingulate | CGI-C | r=0.547, **p=0.035** |
| Rt caudal anterior cingulate | GAF change | r=0.544, **p=0.036** |
| Lt rostral anterior cingulate | CGI-C | r=0.515, p=0.050 |
| Lt pars orbitalis | GAF change | r=0.564, **p=0.028** |
|  | CGI-C | r=0.495, p=0.061 |
| Lt pars triangularis | CGI-C | r=0.639, **p=0.010** |
| Lt pars opercularis | CGI-C | r=0.670, **p=0.006** |
| Rt superior frontal | CGI-C | r=0.532, **p=0.041** |
| Rt cuneus | GAF change | r=0.697, **p=0.004** |
| Lt pericalcarine | GAF change | r=0.613, **p=0.015** |
| Rt pericalcarine | GAF change | r=0.548, **p=0.035** |

Only the regions with significant differences **(at uncorrected p<0.05)** are shown.

The analysis dealt age, sex, MRI scanner and antipsychotic dose with covariates.
