# Supplementary material for: Neural substrates of treatment-resistant schizophrenia and the response to clozapine: A structural MRI study in a clinical setting
Source: PLoS One. 2026 Mar 19;21(3):e0345078. doi: 10.1371/journal.pone.0345078 (PMC13001982; doi:10.1371/journal.pone.0345078)
Supplement: Attachment — Submitted filename: MRICLZinTRS_ResponsetoReviewers_Chiba-u_Masumo.docx [file pone.0345078.s012.docx]

**Revised ms. PONE-D-25-50031: "Neural substrates of treatment-resistant schizophrenia and the response to clozapine A structural MRI study in a clinical setting"**

**Response to the Journal Editor**

Journal Requirements:

When submitting your revision, we need you to address these additional requirements.

Comment 1. Please ensure that your manuscript meets PLOS ONE's style requirements, including those for file naming. The PLOS ONE style templates can be found at

<https://journals.plos.org/plosone/s/file?id=wjVg/PLOSOne_formatting_sample_main_body.pdf> and

[https://journals.plos.org/plosone/s/file?id=ba62/PLOSOne_formatting_sample_title_authors_affiliations.pdf](https://track.editorialmanager.com/CL0/https:%2F%2Fjournals.plos.org%2Fplosone%2Fs%2Ffile%3Fid=ba62%2FPLOSOne_formatting_sample_title_authors_affiliations.pdf/1/010f019add2049ec-c59bbaa0-3ecb-4131-bcb0-8b1db344a987-000000/ET3wqRql2-DF6ERllZtZiTbVie99eDc4e9EV3MeE6lE=239)

**Response:** We have ensured that the manuscript meets the above additional style requirements.

Comment 2. Please provide additional details regarding participant consent. In the ethics statement in the Methods and online submission information, please ensure that you have specified (1) whether consent was informed and (2) what type you obtained (for instance, written or verbal, and if verbal, how it was documented and witnessed). If your study included minors, state whether you obtained consent from parents or guardians. If the need for consent was waived by the ethics committee, please include this information.

If you are reporting a retrospective study of medical records or archived samples, please ensure that you have discussed whether all data were fully anonymized before you accessed them and/or whether the IRB or ethics committee waived the requirement for informed consent. If patients provided informed written consent to have data from their medical records used in research, please include this information.

**Response:** Our study had a retrospective design and we analyzed MRI images that had been scanned for clinical use in the past, but not for research purposes. Regarding study ethics, we note in the manuscript that the study followed the Ethical Guidelines for Medical and Biological Research Involving Human Subjects in Japan. Regarding informed consent, we advertised the study on the two hospitals' websites, providing an opportunity to opt out (described in the manuscript text). We created an independent 'Ethics review' subsection in the Patients and Methods section on page 8.

Comment 3. Thank you for stating the following in the Competing Interests/Financial Disclosure * (delete as necessary) section:

“N.K. reports honoraria from Otsuka Pharmaceutical Co., Sumitomo Pharma Co., Janssen Pharmaceutical., Meiji Seika Pharma Co., Mitsubishi Tanabe Pharma Co., MSD. Y.Od. reports honoraria from Eisai Co., Otsuka Pharmaceutical Co., Meiji Seika Pharma Co. T.N. reports honoraria from Otsuka Pharmaceutical Co., Viatris Inc., Daiichi Sankyo Co., Sumitomo Pharma Co., Meiji Seika Pharma Co., and Yoshitomi Yakuhin Co. He also received research funding from NTT Precision Medicine, Inc. Y.M., Y. Ot., Y.S., T.I., H.S., K.I., H.K., Y.F., Y.H., and M.I. have no conflicts of interest to declare.”

We note that you received funding from a commercial source: “Otsuka Pharmaceutical Co., Sumitomo Pharma Co., Janssen Pharmaceutical., Meiji Seika Pharma Co., Mitsubishi Tanabe Pharma Co. Otsuka Pharmaceutical Co., Viatris Inc., Daiichi Sankyo Co., Sumitomo Pharma Co., Meiji Seika Pharma Co., and Yoshitomi Yakuhin Co”

Please provide an amended Competing Interests Statement that explicitly states this commercial funder, along with any other relevant declarations relating to employment, consultancy, patents, products in development, marketed products, etc.

Within this Competing Interests Statement, please confirm that this does not alter your adherence to all PLOS ONE policies on sharing data and materials by including the following statement: "This does not alter our adherence to PLOS ONE policies on sharing data and materials.” (as detailed online in our guide for authors <http://journals.plos.org/plosone/s/competing-interests>).  If there are restrictions on sharing of data and/or materials, please state these. Please note that we cannot proceed with consideration of your article until this information has been declared.

Please include your amended Competing Interests Statement within your cover letter. We will change the online submission form on your behalf.

**Response:** This study was not supported by any pharmaceutical company. All of the Competing Interest contents are honoraria that are irrelevant to the study but are shown to provide transparency as follows:

“NK reports honoraria from Otsuka Pharmaceutical Co., Sumitomo Pharma Co., Janssen Pharmaceutical., Meiji Seika Pharma Co., Mitsubishi Tanabe Pharma Co., MSD. YOd reports honoraria from Eisai Co., Otsuka Pharmaceutical Co., Meiji Seika Pharma Co. TN reports honoraria from Otsuka Pharmaceutical Co., Viatris Inc., Daiichi Sankyo Co., Sumitomo Pharma Co., Meiji Seika Pharma Co., and Yoshitomi Yakuhin Co. He also received research funding from NTT Precision Medicine, Inc. YM, YOt, YS, TI, HS, KI, HK, YF, YH, and MI have no conflicts of interest to declare. This does not alter our adherence to *PLOS ONE* policies on sharing data and materials.”

Comment 4. We note that your Data Availability Statement is currently as follows:” All relevant data are within the manuscript and its Supporting Information files.”

Please confirm at this time whether or not your submission contains all raw data required to replicate the results of your study. Authors must share the “minimal data set” for their submission. PLOS defines the minimal data set to consist of the data required to replicate all study findings reported in the article, as well as related metadata and methods (<https://journals.plos.org/plosone/s/data-availability#loc-minimal-data-set-definition>).

For example, authors should submit the following data:

- The values behind the means, standard deviations and other measures reported;

- The values used to build graphs;

- The points extracted from images for analysis.

Authors do not need to submit their entire data set if only a portion of the data was used in the reported study.

If your submission does not contain these data, please either upload them as Supporting Information files or deposit them to a stable, public repository and provide us with the relevant URLs, DOIs, or accession numbers. For a list of recommended repositories, please see [https://journals.plos.org/plosone/s/recommended-repositories](https://track.editorialmanager.com/CL0/https:%2F%2Fjournals.plos.org%2Fplosone%2Fs%2Frecommended-repositories/1/010f019add2049ec-c59bbaa0-3ecb-4131-bcb0-8b1db344a987-000000/yLGgESXwipFO-eBammANIcg1iPi82sF88dibHATvHD0=239).

If there are ethical or legal restrictions on sharing a de-identified data set, please explain them in detail (e.g., data contain potentially sensitive information, data are owned by a third-party organization, etc.) and who has imposed them (e.g., an ethics committee). Please also provide contact information for a data access committee, ethics committee, or other institutional body to which data requests may be sent. If data are owned by a third party, please indicate how others may request data access.

**Response:** We think that our manuscript already met these requests.

Comment 5. If the reviewer comments include a recommendation to cite specific previously published works, please review and evaluate these publications to determine whether they are relevant and should be cited. There is no requirement to cite these works unless the editor has indicated otherwise.

**Response:** The two Reviewers' comments did not include a request to add another published article.

**[Note: HTML markup is below. Please do not edit.]**

**Revised ms. PONE-D-25-50031: "Neural substrates of treatment-resistant schizophrenia and the response to clozapine A structural MRI study in a clinical setting"**

**Response to Reviewer#1**

Reviewer #1:

The manuscript investigates cortical volumetric alterations in patients with treatment-resistant schizophrenia (TRS), and explores whether structural MRI findings are associated with response to clozapine (CLZ). The study leverages clinically acquired MRI data (n = 104 patients, plus 109 external healthy controls) and FreeSurfer-based cortical parcellation according to the Desikan–Killiany atlas.

Altough the topic is clinically relevant, the study suffers from significant methodological and interpretative limitations that limit the robustness and novelty of its conclusions. The results are largely negative or nominal, and the discussion sometimes overstates their significance.
Overall, in my opinion the manuscript does not meet the miniml publication standards and should be rejected, moved to a lower impact factor journal, or modified as a brief report with preliminary exploratory nature. I attach below a list of my major concerns.

**Response:** We appreciate the many valuable comments on our work, and we completely agree with all of the comments and accept the Reviewer's concerns. We have made major corrections. New text is presented in red font, and the text exchanged from the Supplementary Material to the main text and vice versa is presented in blue font.

Comment 1. The total number of TRS patients treated with CLZ is small (n=20, with only 12 responders and 8 non-responders). This precludes adequate power for voxel- or region-based analyses, especially when correcting for multiple comparisons. Indeed, none of the reported associations survived FDR correction. Therefore, the interpretation of uncorrected results shoud be presented with strong caution or omitted. Moreover, I advise the authors to explicitly acknowledge the exploratory nature of the analyses and avoid causal or mechanistic interpretations

**Response:** We revised the text to provide a strict statistically significant threshold more clearly and consistently throughout the manuscript. To this end, the following two revisions were made for the comparison of the R-CLZ (n=12) and NonR-CLZ (n=8) groups.

1. Although this comparison was initially planned as the study's main analysis, we were unable to collect a sufficient number of patient cases for this comparison. In the **Results** section, we now state that we collected a smaller sample than initially expected, and we moved the description of this from the main text to the **Supplementary Material**.
2. Throughout the revised manuscript, the results of the analyses on clozapine response (both the between-group comparison and the correlational analysis) with the uncorrected p<0.05 (nominal-level of significance) are described with the negative findings.

These revisions are in the **Abstract**, **Results** and **Discussion** as follows.

**Page 2, Lines 38-40 in the Abstract**

“The correlational analysis of regions related to clozapine responsiveness identified the inferior frontal gyrus (IFG), anterior cingulate cortex (ACC), and occipital cortex at the uncorrected level of significance, but none of these survived the correction for multiple comparisons.”

**Page 21, Lines 281-285 in the Results**

“Since this comparison included a smaller number of patients than initially planned (R-CLZ, n=12; NinR-CLZ, n=8), the results are provided in the **Supplementary Material** and **S6 Table**. Briefly, no significantly different cortical volume region was observed at the statistical threshold after FDR correction in the multiple comparison.”
**Page 24, Lines 321-325 in the Discussion**

“Regarding the responsiveness to CLZ, no regions survived the FDR correction for multiple-group comparisons: we observed that several regions including the inferior frontal gyrus (IFG), ACC, and occipital cortex were related to the CLZ response in the between-group comparison or in the correlational analysis at the uncorrected level for significance (**Table 2, S6, S7 Tables and Fig 4, S1 Fig**).”

**Page 28, Lines 380-385 in the Discussion**

“Of note, since these findings were significance only at the uncorrected level, they should be interpreted with caution. In addition, these subtle differences in our TRS patients may have contributed to this study's overall negative findings regarding the patients' response to CLZ. These regions have been implicated in the pathology of TRS, but we were unable to reach a firm conclusion about this with our present results.”

Comment 2. MRI scans were obtained from five different scanners and protocols, and the healthy control (HC) group was drawn from an external public dataset acquired on different systems. Although the authors included scanner type as a covariate and used ratio normalization, this ususally does not completely correct for inter-scanner variability. Authors should try to run harmonization methods or discuss this issue as a serious limitation that prevents both generalizability and reliable interpretation of data.

**Response:** We agree that the analyses without harmonization were a major study limitation, and we have now described this point in the **Discussion** as follows.

**Page 32, Lines 445-447 in the Discussion**

“This somewhat complicated situation led to difficulty in achieving standard harmonization such as that provided by the ComBat method, thus providing a serious study limitation.”

Comment 3. No correction for antipsychotic dose or age of onset (and therefore duration of illness) is given, altough these parameters are significantly different between groups and could represent strong confounders for structural brain abnormalities. This precludes any reliable inference on outcomes. And the same attains for the sub-classification in clozapine-responders and non-responders, although the reliability of this analysis is already prevented from the very low number of cases.

**Response:** We have added the results controlling for the antipsychotic dose and the patient age at onset in addition to the original variables (i.e., age, sex, MRI) for the comparison of the TRS and non-TRS groups. For the analysis for clozapine response (both the between-group comparison and correlational analysis), we calculated the data including the antipsychotic dose as an additional covariate. These analyses overall obtained almost the same results, which are now presented as novel **S4, S6 and S7 Tables**.

**Page 17, Lines 232-234 in the Results**

“When the patients' age at illness onset and the antipsychotic dose were included as additional covariates, the analysis revealed almost the same results (**S4 Table**).”

**Page 21, Lines 281-283 in the Results**

“Since this comparison included a smaller number of patients than initially planned (R-CLZ, n=12; NonR-CLZ, n=8), the results are provided in the **Supplementary Material** (*2.* *Comparison of cortical volume between the CLZ responders and non-responders*) and **S6 Table**”

**Page 21, Lines 295-296 in the Results**

“These results were almost the same when the analysis included the antipsychotic dose as a covariate (**S7 Table**).”

Comment 4. The operationalization of TRS and the criteria for defining clozapine response/non-response should be described in more detail in the main text (not only in Supplementary Material). The use of clinical global impression (CGI-C) and GAF change over 1 year is reasonable, although not the standard, but the reliability of retrospective classification should be discussed. Moreover, I would avoid to make comparisons between R-CLZ and nR-CLZ patients, given the extremely low sample size in this subclassification and its propensity to false outcomes with this limited power. In my opinion, the section on the comparison between clozapine responders and non-responders should be eliminated as it has poor or no scientific value.

**Response:** We appreciate these comments. As you pointed out, there could be less accuracy concerning the diagnosis of TRS for patients who did not take clozapine since their drug-adherence level were evaluated by their physicians in clinical practice. In contrast, for the TRS patients who were being treated with clozapine, their diagnosis is more valid since physicians are required to confirm the adherence level of their patients prior to the introduction of clozapine. However, even in patients taking clozapine, there might be some problems with the assessments of the clozapine response varying among different physicians. These limitations were derived from our study's retrospective design and were added to the study limitations section. In accord with your instruction, we moved the detailed descriptions of the methodical procedure including the diagnosis and assessments in the **Supplementary Material** back to the main text (**Methods** section).

Regarding the results of the between-group comparison of clozapine responses, we removed the relevant text from the **Results** section and moved it to the **Supplementary Material.**

**Page 3, Lines 38-40 in the Abstract**

“The correlational analysis of regions related to clozapine responsiveness identified the inferior frontal gyrus (IFG), the anterior cingulate cortex (ACC), and the occipital cortex at the uncorrected level of significance, but none of these survived the correction for multiple comparisons.”

**Page 9, Lines 117-159 in the Methods**

“**Patients**

The inclusion criteria were (*i*) status as an inpatient or outpatient who had been diagnosed as having schizophrenia or schizoaffective disorder based on to the Diagnostic and Statistical Manual of Mental Disorders, Fifth edition (DSM-5) and were treated at either of the study hospitals, and (*ii*) having undergone a head MRI examination during the above-described period. We divided the candidate patients into those with TRS and those with non-TRS, based on the operational diagnostic criteria for TRS: the patients who did not show sufficient improvement in psychotic symptoms despite treatment with ≥ 600 mg of the chlorpromazine equivalence (CP-equivalent) of two or more different antipsychotics for ≥ 4 weeks of each trial, and whose Global Assessment of Functioning (GAF) score did not exceed 41 points in the year prior to the introduction of CLZ (i.e., the 'no-response' criteria).

For the TRS patients who had been treated with CLZ, since our study's main purpose was to examine brain regions related to responsiveness to CLZ, we included the MRI images of patients who were scanned within 2 months before the CLZ commencement. For the other patients (i.e., the non-TRS patients and the patients with TRS but medicated with non-CLZ agents), we collected their MRI images if their medication was stable for ≥ 1 month prior to the MRI examination. **Figure 1** is the flow chart of the study's analyses.

We excluded patients: (*i*) whose reason for CLZ introduction met the 'intolerance to antipsychotics' criteria, (*ii*) who had psychiatric disorder(s) other than schizophrenia or schizoaffective disorder, (*iii*) with significant organic brain disease or epilepsy, or (*iv*) who had a serious physical disease, and (*v*) patients who were considered inappropriate for the study by their physicians.

**Assessments**

Indicators at the time of head MRI imaging

The patients' clinical information was collected from their medical records: sex, age at the MRI examination, age at disease onset, and number of hospitalizations prior to the MRI examination. The antipsychotic medication each patient was taking at the time of the MRI examination was also extracted, and the medication doses were converted to the CP-equivalent dose (27, Gardner et al., 2010; 28, Inada and Inagaki, 2015).

Indicators of the patients' responsiveness to CLZ

The following psychopathological assessments were administered only to the patients who were being treated with CLZ: the length of time before the introduction of CLZ treatment (defined as the length of time from the timepoint of the establishment of TRS to the timepoint of CLZ induction), the Brief Psychiatric Rating Scale (BPRS) (29, Kolalowska 1976), the GAF scale, and the Clinical Global Impression-Severity (CGI-S) in the month prior to the CLZ introduction; in addition, the GAF, CGI-S, and Clinical Global Impression-Change (CGI-C) scale at 1 year after the introduction of CLZ. The duration of illness was calculated from the onset of illness to the CLZ induction.

Regarding the patients' responsiveness to CLZ, 'responder' was defined as a ≥20-point change in the patient's GAF score from baseline to 1 year of treatment. The patients whose GAF score changes were <20 points were classified as non-responders. These were obtained by the patients' physicians as routine clinical practice.”

**Page 14, Lines 197-209 in the Results**

“**Demographic information of the TRS and non-TRS groups**

Magnetic resonance images were extracted from 104 patients (TRS patients, n=40; non-TRS patients, n=64). A total of 109 magnetic resonance images of healthy Japanese individuals were extracted from the DecNef-Project Consortium dataset. As shown in **Table 1**, the sex and age at the MRI examination did not differ among the TRS, non-TRS, and HC groups. The age at illness onset in the TRS group was significantly younger than that in the non-TRS group. The antipsychotic dose at the MRI examination in the TRS group was significantly higher than that in the non-TRS group (p<0.001).

Within the TRS group, 20 patients (12 females, eight males) had been under treatment with CLZ. Although there were no significant differences in demographic measures between the patients under subsequent CLZ treatment and the patients being treated with other antipsychotics, significant differences were observed for treatment-related measures: the number of hospital admissions and the antipsychotic dose at the MRI examination in the patients treated with CLZ were significantly higher than those in the patients treated with non-CLZ antipsychotics (both p<0.05) (**S2 Table)**.”

**Page 21, Lines 281-285 in the Results**

“Since this comparison included a smaller number of patients than initially planned (R-CLZ, n=12; NonR-CLZ, n=8), the results were provided in **Supplementary Material** and **S6 Table**. Briefly, no significantly different cortical volume region was observed at the statistical threshold after FDR correction in the multiple comparison.”

**Page 31, Lines 429-434 in the Discussion**

“Regarding the diagnosis of TRS, the validity of patients' TRS diagnoses might have been less accurate since the patients' adherence to medication were confirmed only by the physicians' observations: specifically, this is true of the patients who were classified as having TRS but were not administered CLZ. This inconsistent data collection and somewhat rough diagnosis circumstances hindered rigorous analyses.”

**Page 32, Lines 440-442 in the Discussion**

“Their clinical scores might also have been influenced by the assessments having been conducted by different physicians as well.”

Comment 5. The central hypothesis that cortical morphology correlates with the delay before CLZ introduction was not confirmed. This negative finding, which contradicts the stated rationale, should be given greater prominence in the abstract and discussion, rather than being overshadowed by nominal findings on other regions. Moreover, it is not clear on which literature base the authors posited this hypothesis. And in general, the introduction is not useful to capture the rationale of the study or a clear neurobiologically-based hypothesis, other than assuming that there are few previous reports on the issue. And the strong claim on the evaluation of biomarkers related to clozapine therapy should be mitigated, given than only 20 patients of the sample were under clozapine.

**Response:** In the revised manuscript, we have more clearly prioritized the analysis of the length of time before clozapine introduction in **Abstract** and **Discussion**. A more explanation of the potential importance of this indicator was added to the **Introduction** section. Our insufficient analysis due to the small number of patients and the failure to reach a conclusion on this indicator are clearly described in the **Discussion**. These corrections are as follows.

**Page 3, Lines 31-33 in the Abstract**

“We then investigated brain regions related to both responsiveness to clozapine and the duration between the TRS designation and the introduction of clozapine.”

**Page 4, Lines 45-46 in the Abstract**

“The lack of correlations between the length of time prior to clozapine introduction and cortical regions might have been derived by insufficient statistical power, thus necessitating further research.”

**Page 6, Lines 71-82 in the Introduction**

“This problem is very relevant in clinical practice since the optimal timing for the introduction of CLZ to achieve a better prognosis has not been established. Most patients with schizophrenia first receive CLZ several years after they meet the TRS criteria mentioned above. It is possible that a prolonged exposure to glutamine neurotoxicity or dopamine supersensitivity could negatively affect patients' brain substrates, but the mechanisms underlying this are not fully understood. Indirect findings in patients have indicated that the duration of relapse episodes or the treatment intensity after a relapse of psychosis could lead to deficits in brain volumes [19–21, Guo et al., 2015]. These data also suggested that a longer period of time before the introduction of CLZ treatment could have negative impacts on patients' brains due to the development of unstable psychosis and/or the inability of high-dose non-CLZ antipsychotic medication to control psychosis. However, our search of the relevant literature identified no reports of potential links between neuronal substrates in TRS patients and the length of time prior to their commencement of treatment with CLZ.”

**Page 24, Lines 325-326 in the Discussion**

“In addition, no cortical region was related to the length of time prior to CLZ introduction even at the uncorrected level, a finding that does not support our study hypothesis.”

**Page 28, Lines 393-394 in the Discussion**

“This result is partly consistent with those of earlier reports and supports the clinical importance of the length of time prior to the introduction of CLZ.”

In relation to the above change, the following paper was added to the **Reference** list.

21. Guo, J.Y., Huhtaniska, S., Miettunen, J., Jääskeläinen, E., Kiviniemi, V., Nikkinen, J., Moilanen, J., Haapea, M., Mäki, P., Jones, P.B., Veijola, J., Isohanni, M., Murray, G.K., 2015. Longitudinal regional brain volume loss in schizophrenia: Relationship to antipsychotic medication and change in social function. Schizophr. Res. 168, 297-304.

Comment 6. The discussion is in large part speculative, as it repeatedly refers to “involvement” of IFG, ACC, and occipital cortex in CLZ response, yet all findings are at p < 0.05 uncorrected. These findings should be clearly labelled as exploratory and interpreted accordingly, with great caution and no causal inferences.

**Response:** We have changed the description of the results with uncorrected p-values <0.05 into negative findings throughout the manuscript. Parts of the **Discussion** text regarding the regions identified in the analysis of clozapine response (IFG, ACC and occipital cortex) were largely deleted and are now described as requiring caution for the interpretation as follows.

**Page 3, Lines 35-38 in the Abstract**

“However, there was no significant difference in the brain regions between the TRS and non-TRS groups: compared to the non-TRS group, the TRS group showed smaller volumes in a wider range of brain regions only at the uncorrected level.”

**Page 4, Lines 43-45 in the Abstract**

“These results suggest that the IFG, ACC, and occipital cortex might be responsible for the treatment response to clozapine, but this must be interpreted cautiously due to the small sample size.”

**Page 24, Lines 321-325 in the Discussion**

“Regarding the responsiveness to CLZ, no regions survived the FDR correction for multiple-group comparisons: we observed several regions including the inferior frontal gyrus (IFG), ACC, and occipital cortex were related to the CLZ response in the between-group comparison or in the correlational analysis at the uncorrected level for significance (**Table 2, S6, S7 Tables and Fig 4, S1 Fig**).”

**Page 27, Lines 375-385 in the Discussion**

“Alteration in the ACC was observed in several TRS neuroimaging studies: a smaller regional volume or cortical thickness in TRS patients [22,42,46] or CLZ non-responders [22,25], and a higher glutamate or glutamate-glutamine (Glx) level in patients with TRS [47-49] or ultra-treatment-resistant schizophrenia (UTRS) [50]. Other studies have demonstrated that the IFG and occipital cortex in TRS patients showed a regional volume reduction or cortical thinness compared to healthy controls [22,24,35-37,39,42,46] or non-TRS patients [24,25,38,40,41].Of note, since these findings were significance only at the uncorrected level, they should be interpreted with caution. In addition, these subtle differences in our TRS patients may have contributed to this study's overall negative findings regarding the patients' responses to CLZ. These regions have been implicated in the pathology of TRS, but we were unable to reach a firm conclusion about this with our present results.”

Comment 7. The choice to analyze cortical volume ratios rather than absolute values or thickness measures is unconventional and not well justified. The authors should clarify why this approach was preferred and discuss its possible drawbacks.

**Response:** As a method for the correction necessary for the use of different MRI scanners, the calculation of the volume ratio has gained consensus to some degree, but the biggest problem with this method is that in the case of patients/disease with broader cortical regions affected, a calculated ratio could cancel the actual difference in its corresponding regional volume. In our analysis of the TRS vs. non-TRS data, our observations of a lack of a significant difference in the volume ratio analysis but the presence of statistical significance in the volume analysis were likely an example of this. This point was added to the **Discussion** and is noted in the study limitations section as follows.

**Page 26, Lines 346-351 in the Discussion**

“Our TRS group exhibited middle- to-large effect sizes of smaller cortical volume in multiple areas compared to the HC group in the volume analysis (**S5 Table**). The reason for the almost complete absence of a significant region in the volume ratio analysis (**S3 Table**) is most likely that the whole-brain volume (ICV) was decreased, resulting in the attenuation of an actual decrease in a regional volume in the patient groups. It thus appears that the brain structural alterations in our TRS patients spread widely to broad cortical areas, which is consistent with the findings of earlier investigations.”

Comment 8. The manuscript would benefit from linguistic editing to improve clarity and conciseness. Several sections are redundant or overly descriptive. Figures and tables are informative but should highlight key results more effectively (e.g., provide effect sizes and FDR-adjusted p-values in the main text).

**Response:** We had a native English speaker edit the manuscript. As shown in our responses to other comments, we shortened the **Discussion** section by deleting the descriptions concerning the results with nominal-level significance. We also reconstructed the figures and tables by moving those describing minor results to the Supplementary Materials. We added several statistical values for the key analyses to the main text in the **Results** section as follows.

**Page 17, Lines 222-224 in the Results**

“At the uncorrected level, however, this analysis identified only one region, the left frontal pole, the volume of which was significantly larger in the TRS group compared to the non-TRS group (*F*=4.610, p=0.034, Cohen's *d*=0.190).”

**Page 17, Lines 227-232 in the Results**
“… the bilateral lateral frontal cortices (the left caudal middle frontal, bilateral rostral middle frontal, and right superior frontal cortices), the medial frontal cortices (left caudal anterior cingulate [cACC]), the bilateral temporal cortices (the bilateral bank of the superior temporal sulcus [STS], and the right transverse temporal, bilateral superior temporal, and bilateral middle temporal cortices), the right lateral occipital, and the medial occipital cortex (i.e., cuneus) (The right cuneus has the largest Cohen's *d* (0.508) with *F*=7.643, p=0.007, as shown in **S3 Table)**.”

**Page 19, Lines 258-260 in the Results**
“Most of these regions exhibited middle-to-large effect sizes (Cohen's *d-*value >0.40), and the region with the greatest effect size was the left rACC in both the TRS group (*d* =0.988) and the non-TRS group (*d* =0.946) (**S5 Table**).”

**Page 19, Lines 262-263 in the Results**

“The region with the greatest effect size (*d*=0.422) was the right paracentral cortex.”

Comment 9. Authors should clarify the time relationship between MRI acquisition and the initiation of clozapine treatment (was the MRI always before CLZ introduction or any other else?). The ethics section should specify the form of consent (written vs. opt-out) for both institutions.

**Response:** Our study included only MRI images scanned within 2 months prior to the clozapine introduction.

Concerning the ethical requirement, the study complies with the Ethical Guidelines for Medical and Biological Research Involving Human Subjects in Japan. We analyzed MRI images that were examined for clinical practice in the past. These guidelines instruct researchers to announce the study information to subjects and to offer an opportunity to decline study participation. We now describe this as follows.

**Page 8, Lines 112-115 in the Methods**

“Regarding the requirement for patients' informed consent, the detailed information of the study was provided on our department's website and gave the patients an opportunity to consider participation in the study on an opt-out basis. The study followed the Ethical Guideline for Medical and Biological Research Involving Human Subjects in Japan.”

**Page 9, Lines 127-131 in the Methods**

“For the TRS patients who had been treated with CLZ, since our study's main purpose was to examine brain regions related to responsiveness to CLZ, we included the MRI images of patients who were scanned within 2 months before the CLZ commencement. For the other patients (i.e., the non-TRS patients and the patients with TRS but medicated with non-CLZ agents), we collected their MRI images if their medication was stable for ≥ 1 month prior to the MRI examination.”

**Revised ms. PONE-D-25-50031: "Neural substrates of treatment-resistant schizophrenia and the response to clozapine A structural MRI study in a clinical setting"**

**Response to Reviewer#2**

Reviewer #2:

This study investigates grey matter volumen correlates to TRS and CPZ repsonse in patients with schizophrenia. The study questions are interesting and clinically relevant, and the authors aim to answer the research questions based on pragmatic solutions on data-quality, sample-size, methodological complexity a.o.. There are however a range of major and minor concerns about the study:

**Response:** We appreciate your insightful comments, and we have made text revisions in accord with the comments. New text is presented in red font, and text that has been moved from the **Supplementary Material** to the main text and vice versa is presented in blue font.

Comment 1. It is a major concern that the study and data acquisition was not designet for these hypothesis, but was derived from a pragmatic pooling of data from 7 different scanners with vital differences in acquisition parameters such as Field Strenght, REp. time, Inversion, flip angle etc. (according to Supplem. Table S1). The authors state as imitations that harmonization attempts failed, hence they used the very crude correction for scanner type as covariate. As the major results reported is groupdiffernce between patients and controls, where the MRI data for controls were from a very different public dataset, the results are unfortunately questionable. What could validate the results more would be transparence about an extensive examination of data, including an option of visual inspection of e.g. scatterplots etc. by reader and reviewers. However, as there are more major considerations, such as a lack of power calculation, resulting in a far to small sample (8 non-responsers vs 12 responding CPZ patients when examining groupdifferences) to meaningfully answer the relevant research questions. The authors may - with the caveats from the pooling of data from different scanners - meaningfully investigate differences between controls and patients, but further subgrouping of patients will reduce the degrees of freedom to a point where analyses cannot be evaluated as sound / nor results as robust with this sample size. It is a huge concern with yet another MRI study presenting non-robust results on insufficient data of limited quality. The authors state (line 228-229) that they provide several insights into the relationship between brain structure and responsiveness to CPZ - but this conclusion is not valid. At best, the study may be hypothesis generating due to the pragmatic design and major concerns.

**Response:** We agree with these serious concerns, particularly the different MRI scanners used for the patients in clinical practice, the use of an open database of healthy controls, and the small sample size for the analysis of clozapine responses. We revised the following two points to increase the methodological transparency even slightly: (1) Details of the methods including the collection of MRI data and the patients' clinical information were moved back to the **Methods** section from the **Supplementary Material**. (2) The results of the between-group comparison of responses to clozapine (including only 20 patients) were moved to the **Supplementary Material**.

Regarding the text that you mention at lines 228-229, we have rewritten this part of the text.

**Page 9, Lines 117-180 in the Methods**

“**Patients**

The inclusion criteria were (*i*) status as an inpatient or outpatient who had been diagnosed as having schizophrenia or schizoaffective disorder based on to the Diagnostic and Statistical Manual of Mental Disorders, Fifth edition (DSM-5) and were treated at either of the study hospitals, and (*ii*) having undergone a head MRI examination during the above-described period. We divided the candidate patients into those with TRS and those with non-TRS, based on the operational diagnostic criteria for TRS: the patients who did not show sufficient improvement in psychotic symptoms despite treatment with ≥ 600 mg of the chlorpromazine equivalence (CP-equivalent) of two or more different antipsychotics for ≥ 4 weeks of each trial, and whose Global Assessment of Functioning (GAF) score did not exceed 41 points in the year prior to the introduction of CLZ (i.e., the 'no-response' criteria).

For the TRS patients who had been treated with CLZ, since our study's main purpose was to examine brain regions related to responsiveness to CLZ, we included the MRI images of patients who were scanned within 2 months before the CLZ commencement. For the other patients (i.e., the non-TRS patients and the patients with TRS but medicated with non-CLZ agents), we collected their MRI images if their medication was stable for ≥ 1 month prior to the MRI examination. **Figure 1** is the flow chart of the study's analyses.

We excluded patients: (*i*) whose reason for CLZ introduction met the 'intolerance to antipsychotics' criteria, (*ii*) who had psychiatric disorder(s) other than schizophrenia or schizoaffective disorder, (*iii*) with significant organic brain disease or epilepsy, or (*iv*) who had a serious physical disease, and (*v*) patients who were considered inappropriate for the study by their physicians.

**Assessments**

Indicators at the time of head MRI imaging

The patients' clinical information was collected from their medical records: sex, age at the MRI examination, age at disease onset, and number of hospitalizations prior to the MRI examination. The antipsychotic medication each patient was taking at the time of the MRI examination was also extracted, and the medication doses were converted to the CP-equivalent dose (27, Gardner et al., 2010; 28, Inada and Inagaki, 2015).

Indicators of the patients' responsiveness to CLZ

The following psychopathological assessments were administered only to the patients who were being treated with CLZ: the length of time before the introduction of CLZ treatment (defined as the length of time from the timepoint of the establishment of TRS to the timepoint of CLZ induction); the Brief Psychiatric Rating Scale (BPRS) (29, Kolalowska 1976); the GAF scale, and the Clinical Global Impression-Severity (CGI-S) in the month prior to the CLZ introduction; in addition, the GAF, CGI-S, and Clinical Global Impression-Change (CGI-C) scale at 1 year after the introduction of CLZ. The duration of illness was calculated from the onset of illness to the CLZ induction.

Regarding the patients' responsiveness to CLZ, 'responder' was defined as a ≥20-point change in the patient's GAF score from baseline to 1 year of treatment. The patients whose GAF score changes were <20 points were classified as non-responders. These were obtained by the patients' physicians as routine in the clinical practice.

**Image capture and analysis**

The patients' three-dimensional T1-weighted images were obtained with the use of five different MRI scanners at Chiba University Hospital (protocol MRI-1, n=55; MRI-2, n=20; MRI-3, n=21; MRI-4, n=7) and Asahi General Hospital (MRI-5, n=1). (**S1 Table**) The structural MRI data were acquired by covering the whole brain with a sagittal 3D-magnetization-prepared gradient echo sequence. The imaging parameters are in **S1 Table**.

For the MRI images of healthy controls (HCs), we leveraged the dataset of brain MRI images of healthy Japanese adults in the DecNef-Project Consortium (<http://www.cns.atr.jp/decnefpro/>). A total of 109 images (protocol MRI-6, n=98; MRI-7, n=11; **S1 Table**) were selected so that the HCs' ages and sexes were matched with those with the patients.

We excluded manually obtained images in which the entire brain was not imaged appropriately or showed clear artifacts. The image analysis was performed using the FreeSurfer 7.1.1 program (https://surfer.nmr.mgh.harvard.edu/). We also excluded images with a voxel registration error by conducting a visual quality assessment. The cortical volume per brain region was calculated based on the Desikan-Killiany (DK) atlas cited below. We did not compare the cortical thickness values, but we compared the groups' cortical gray matter-volume ratios (i.e., the ratio of the regional volume/intra cranial volume [ICV]; we used the estimated total intracranial volume [eTIV] in FreeSurfer), which are more appropriate for the comparisons of structures between the patient groups and HC group since the images were obtained with five different MRI scanners (30, Backhausen et al., 2022). We used the DK atlas to visualize the results (31, Desikan et al., 2006).”

**Page 21, Lines 281-285 in the Results**

“Since this comparison included a smaller number of patients than initially planned (R-CLZ, n=12; NonR-CLZ, n=8), the results are provided in the **Supplementary Material** and **S6 Table**. Briefly, no significantly different cortical volume region was observed at the statistical threshold after FDR correction in the multiple comparison.”

**Page 24, Lines 326-330 in the Discussion**

“Although we identified significant structural declines in the TRS and non-TRS groups, the results of this study also indicated that the negative findings concerning the regions related to the patients' responses to CLZ and the length of time prior to CLZ introduction in the patients with TRS were likely to have been influenced by the study's small sample size, requiring a cautious interpretation of our findings.”

Comment 2. Another major concern is the attempt to link MRI GMV data to CPZ response. First, it is very uncertain that any difference has an MRI-signal, as the patients were not antipsychotic naive before start CPZ. Secondly, it is not proper to present this mixture of uncorrected (i.e. "nominal level of significance" as in line 182) and corrected results, particularly with the huge number of correlation analyses - a method known for producing to many false positive results. If the authors - after correction for multiple analyses - find that no GM regions survived control when comparing CPZ responders to nonresponders, that is the result which has to be clearly stated in eg. the abstract. It is not, to the contrary it appears like there is a difference, which is not trustworthy.

**Response:** The text now clearly interprets the findings with uncorrected p-values <0.05 as negative results throughout the manuscript. The parts related to this point are as follows.

**Page 3, Lines 35-42 in the Abstract**

“However, there was no significant difference in the brain regions between the TRS and non-TRS groups: compared to the non-TRS group, the TRS group showed smaller volumes in a wider range of brain regions only at the uncorrected level. The correlational analysis of regions related to clozapine responsiveness identified the inferior frontal gyrus (IFG), anterior cingulate cortex (ACC), and the occipital cortex at the uncorrected level of significance, but none of these survived the correction for multiple comparisons. No relationship between any cortical region and the length of time prior to clozapine introduction was observed.”

**Page 24, Lines 317-325 in the Discussion**

“Compared to the non-TRS patients, the TRS patients did not exhibited significantly different brain regions at the FDR correction. However, we observed smaller cortical volumes in the lateral and medial frontal temporal cortices and the occipital region in the TRS patients compared to the non-TRS patients at an uncorrected level for significance (**S3, S4 Table and Fig 2)**.

Regarding the responsiveness to CLZ, no regions survived the FDR correction for multiple-group comparisons: we observed that several regions including the inferior frontal gyrus (IFG), ACC, and occipital cortex were related to the CLZ response in the between-group comparison or in the correlational analysis at the uncorrected level for significance (**Table 2, S6, S7 Tables and Fig 4, S1 Fig**).”

Comment 3. The use of unusual words and terms, which are a bit uncorrect, and claims which demonstrate lack of clinical understanding (such as line 36 "broader brain regions"; line 153 "MRI-system" (usually we term it scanner / scannersites); line 165-167 are not understandable . do the authors correct for different scanners or not in the analyses?; line 301 "cortex was less damaged"; line 286-287, claiming that hallucinations, negative symptoms and cognitive dysfunction is a "potential core psychopathology of TRS" (these are decisive symptoms for all patients with schizophrenia??).

**Response:** We appreciate these instructive comments and have made corrections based on them. Regarding lines 165-167, we intended this as you noted: we reframed this part of the text as follows. The text at lines 286-287 were deleted from the revised manuscript.

**Page 3, Lines 36-38 in the Abstract**

“…compared to the non-TRS group, the TRS group showed smaller volumes in a wider range of brain regions only at the uncorrected level.”

**Page 12, Line 175-179 in the Methods**

“We did not compare the cortical thickness values, but we compared the groups' cortical gray

matter-volume ratios (i.e., the ratio of the regional volume/intra cranial volume [ICV]; we used the estimated total intracranial volume [eTIV] in FreeSurfer), which are more appropriate for the comparisons of structures between the patient groups and HC group since the images were obtained with five different MRI scanners (30, Backhausen et al., 2022).”

**Page 13, Lines 187-191 in the Methods**

“For the analysis of brain cortical volumes, we performed an analysis of covariance (ANCOVA) with age, sex, and the MRI scanner as covariates for the two- and three-group comparisons since the patients' images were obtained with five different MRI scanners. A partial correlational analysis with age, sex, and the MRI scanner as covariates was performed to investigate the relationship between each clinical indicator and the cortical-volume ratio of each segment.”

**Page 17, Lines 220-222 in the Results**

“As shown in **S3 Table**, the comparison of cortical volume ratios between the TRS and non-TRS groups controlling for age, sex, and the MRI scanner did not detect a significant difference at the statistical threshold after FDR correction.”

**Page 19, Lines 248-251 in the Results**

“The ANCOVA with age, sex, and the MRI scanner as covariates revealed significant differences in only five regions at the FDR-corrected threshold: the bilateral medial orbitofrontal cortex (mOFC), the left rostral anterior cingulate cortex (rACC), the left frontal pole, and the right superior frontal gyrus.”

**Page 19, Lines 263-267 in the Results**

“Regarding the difference between the results of the analyses with and without the MRI scanner included as a covariate, we suspect that since assigned covariates for MRI scanners were separated by groups (i.e., designated 'MRI-1'-'MRI-5' to the patients and 'MRI-6' and 'MRI-7' to the HCs, respectively in **S1 Table**), any actual differences in volumes between groups likely disappear by correcting MRI scanners.”

**Page 32, Line 443-449 in the Discussion**

“A fourth study limitation is that the MRI images were obtained with five different MRI scanners. For the group of healthy controls, we turned to the open dataset of healthy participants based on MRI findings and on protocols that differed from those used for our patients. This somewhat complicated situation led to difficulty in achieving standard harmonization such as that provided by the ComBat method, thus providing a serious study limitation. We thus used the relative ratio of volume (regional volume per ICV) and the MRI scanners as covariates for the comparisons with the healthy controls' data and several analytic procedures.”

Comment 4. Lack of clarity in reporting the study design, materials and methods is a huge obstacle for transparency in this study. The methods reported in supplements needs to go to the main text, and it would be beneficial with a illustration of the analyses plan, and which data came from which group / subgroup / timepoint, as it is hard to deciphre in the current format. Further, it is unclear in Table 2 that you actually appear to have tested every clinical measure at against coorticsl regions, but in some it is delta (change), but not in others etc.

**Response:** We apologize for the confusion. In accord with your comment, we moved the detailed description of methodological procedures in the **Supplementary Material** to the main text (Methods section). To further clarify our analysis procedure, we created a novel **Figure 1**.

Regarding the correlational analysis, we analyzed the correlations between every region and each clinical parameter (GAF at baseline, length of time prior to CLZ introduction, age at onset, age at TRS designation, and age at CLZ introduction, in addition to GAF change and CGI-C) as you point out, and we mention only the regions related to the parameter with at least uncorrected p<0.05 in **Table 2** of the original manuscript (these analyses detected several regions related to only GAF change and CGI-C as shown in **Table 2**). However, given your comment and our identification of an error in this revision, we revised the Table to make it more clear and added the results.

In relation to these corrections, the novel **Figure 1** was registered.

**Page 10, Lines 131-132 in the Methods**

“**Figure 1** is the flow chart of the study's analyses.”

**Page 23, Lines 307-311 in the Results**

“There was no measure prior to the commencement of CLZ treatment (i.e., age at disease onset, age at the TRS diagnosis, age at the CLZ commencement, or the length of time before the introduction of CLZ) that showed a significant relationship with any cortical volume in the CLZ-treated patients at the FDR-corrected threshold, and at the uncorrected level only the patients' age at disease onset was related to two regions (left cACC, r=－0.503, p=0.047; left paracentral, r=0.502, p=0.048).”

Comment 5. Minor concerns would be the lenght of the discussion, with a lot of redundance, such as line 22-225 repeated in 237-239, and it appears unstructured, with far too much speculation on results which cannot justify such discussions.

**Response:** We shortened and reframed the **Discussion** section. The original text at lines 222-225 and lines 237-239 described different outcomes (the former is the result of the analysis of clozapine responses and the latter is that of the analysis comparing the TRS and non-TRS groups), indicating no duplicated descriptions. However, we carefully checked the **Discussion** and eliminated redundant sentences (as shown in our responses to other comments).

Comment 6. A major revision, using the principle of Occams razor, where the authors refrain from underpowered analyses, not overreport results, and instead simplify the study questions and enhance clarity and transparency of the pragmatic and preliminary hypothesis-generating results would result in an article of much higher scientific quality.

**Response:** We greatly appreciate the valuable comments and have made revision by keeping these comments in mind.
