# Supplementary material for: Neural substrates of treatment-resistant schizophrenia and the response to clozapine: A structural MRI study in a clinical setting
Source: PLoS One. 2026 Mar 19;21(3):e0345078. doi: 10.1371/journal.pone.0345078 (PMC13001982; doi:10.1371/journal.pone.0345078)
Supplement: Attachment — Submitted filename: MRICLZinTRS_ResponsetoReviewers_Chiba-u_Masumo_auresp_2.docx [file pone.0345078.s013.docx]

**Revised ms. PONE-D-25-50031R2: "Neural substrates of treatment-resistant schizophrenia and the response to clozapine A structural MRI study in a clinical setting"**

**Responses to Reviewer 1**

Reviewer 1:

The authors have addressed most of my concerns and appropriately toned down their conclusions. While important methodological limitations remain, the manuscript now presents a clearer and more transparent exploratory analysis that is suitable for publication in PLOS ONE.

**Response:** Thank you. We appreciate your helpful input and review of our manuscript.

**Revised ms. PONE-D-25-50031R2: "Neural substrates of treatment-resistant schizophrenia and the response to clozapine A structural MRI study in a clinical setting"**

**Responses to Reviewer 2**

Reviewer 2:

Comment 1. The authors have not adressed my concerns sufficiently. The first major concern reg. data acquisition from a pragmatic pooling of data from 7 different scanners with vital differences in acquisition parameters where authors state that harmonization attempts failed, plus the MRI data for controls were from a very different public dataset. I suggested
" more transparence about an extensive examination of data, including an option of visual inspection of e.g. scatterplots etc. by reader and reviewers", but the only action taken by the authors is to move text from supplementary to main text. I would like a thorough examination of how attempts to harmonize was failing, including the results as shown by eg.scatterplots etc. - if not, the conclusions cannot be regarded as valid.

**Response:** We are very sorry that we did not adequately address the reviewer’s concerns in our previous revision of our manuscript. In this revised version, we present additional scatter plot data showing individual volume distributions. In order to enhance the data transparency and to provide a resource that could be clearly interpreted for the potential effects of different MRI scanners on brain volume, the overall trend showing the distribution in brain volume (new **S1 Figure**) and the analysis of the effect of each MRI scanner on regional volume in each group (new **S2 Figure**) are now presented. A detailed explanation of this analysis is given in the Supplementary Materials as shown below. As a result of this analysis, as the reviewer pointed out, we conclude that specific scanner(s) could potentially affect regional volume for subjects, but our attempt to apply volume-ratio analyses could have attenuated the effect of MRI scanners on between-group differences.

**Page 20, Lines 266-269 in the Results**

“A further analysis of the potential effects of different MRI scanners on the between-group comparisons was conducted; the results are shown in the **Supplementary Materials** (*2. Potential effects of MRI scanners within each group)*.”

**The 2^nd^ section of the Supplementary Material**

“***2. Potential effects of MRI scanners within each group***

Since the present study did not apply harmonization (ComBat) to correct for potential scanner effects, we examined whether difference among MRI scanners could have influenced the findings.

First, in the patient group, whole-brain volume (“BrainSegVol”) appeared unlikely to be influenced by the five MRI scanners (**S1A Fig**). Similarly, in the HC group, there were no clear differences in the brain-volume distributions between the two scanners (**S1B Fig**). However, because the patient and HC groups differed in the MRI scanners used, we further examined whether scanner effects could influence the between-group comparison of brain volume. As an example, we show results for the left rACC **(S2 Fig),** which exhibited the largest difference among the three groups in the volume-ratio analyses (*F*=32.595, p=1.0×10^-10^: **S5 Table**). In the volume-level comparison, HC volume appeared slightly larger than TRS volume, consistent with the statistical significance by three-group ANOVA (*F*=8.580, p<0.001; TRS<HC, p<0.001, Bonferroni corrected *post-hoc* test) (**S2A Fig**). For eTIV, on the other hand, it is likely that the HC had slightly smaller volume than the non-TRS, which was supported by the three-group ANOVA (*F*=10.007, p<0.001; nonTRS>HC, p<0.001, Bonferroni correct *post-hoc* test) (**S2B Fig**). Notably, within the HC group, participants scanned on MRI-7 showed a trend toward smaller volumes. Together, these observations suggest that volume-level comparisons (particularly comparison vs. HCs drawn from an open dataset) could be influenced by scanner differences, because a global trend toward larger volumes in schizophrenia relative to HCs would be unexpected. When we instead analyzed volume ratios, the separation between HC and patient groups became clearer (**S2C Fig**; also see the **Results** section), and the MRI-7-specific trend was no longer apparent. Thus, although scanner-related differences seemed minimal within the patient and HC groups considered separately (**S1 Fig**), scanner effects may still have influenced between-group comparisons. The volume-ratio approach may help attenuate such effects and may better reflect true between-group differences.”

In relation to these changes, we added two supplemental figures to **Supporting information**

**S1 Fig. Scatter plots showing the relationship between brain volume and age by different MRI scanners for (A) patients and (B) healthy controls.**

**S2 Fig. Scatter plots showing volume distributions in (A) the left rACC and (B) eTIV, and (C) rACC volume ratio (derived by A/B) by different MRI scanners in the TRS, non-TRS and HC groups.** The horizontal line represents the mean of each group.

Comment 2. My second concern "a lack of power calculation, resulting in a far to small sample (8 non-responsers vs 12 responding CPZ patients when examining groupdifferences) to meaningfully answer the relevant research questions. The authors may - with the caveats from the pooling of data from different scanners - meaningfully investigate differences between controls and patients, but further subgrouping of patients will reduce the degrees of freedom to a point where analyses cannot be evaluated as sound / nor results as robust with this sample size." was solved by authors by moving the subgroup analyses to the supplementary and change some text - but still in abstract authors write predominantly about this subject - in results: "The correlational analysis of regions related to clozapine responsiveness identified the inferior frontal gyrus (IFG), anterior cingulate cortex (ACC), and the occipital cortex at the uncorrected level of significance, but none of these survived t he correction for multiple comparisons" and in Conclusion "These results suggest that the IFG, ACC, and occipital cortex might be responsible for the treatment response to clozapine....". It is very difficult to infer from what could have been a result in case of more power, but here I truly find this to be scientifically unsound, and not just something that be framed differently by small reformulaitons. I cannot recommend accept without thorough rewriting from a much more humble perspective on what is possible to infer by this study design and underpowered samplesize.

**Response:** We agree with the reviewer. In the revised manuscript, we further toned down our interpretation of this study, and we now describe our results as completely negative. We changed the following portions in the **Abstract** and **Conclusion** section accordingly.

**Page 3, Line 38-Page, 4, Line 39 in the Abstract**

“The correlational analysis of regions related to clozapine responsiveness did not identify any region that survived the correction for multiple comparisons.”

**Page 4, Lines 41-42 in the Abstract**

“Overall, these results failed to identify the cortical region responsible for the treatment response to clozapine.”

**Page 33, Lines 457-459 in the Conclusion**

“Differences in cortical structure between CLZ responders and non-responders were not observed at a stringent statistical threshold; however, several regions (IFG, ACC, and occipital cortex) were identified in between-group and correlational analyses at an uncorrected level.”
